# Binding free energy analysis of galectin‐3 natural ligands and synthetic inhibitors

**DOI:** 10.1002/pro.70143

**Published:** 2025-05-22

**Authors:** Luke Newman, Valerie Vaissier Welborn

**Affiliations:** ^1^ Department of Chemistry Virginia Tech Blacksburg Virginia USA; ^2^ Macromolecules Innovation Institute Virginia Tech Blacksburg Virginia USA

**Keywords:** alchemical, AMOEBA, binding free energy, galectin, galectin‐3, inhibitor, ligand, molecular dynamics

## Abstract

Galectin‐3–ligand complexes are characterized by halogen, σ‐hole bonds, hydrogen bonds, cation‐π and CH‐π interactions. Here, we model these non‐covalent interactions with the AMOEBA polarizable force field and conduct an absolute binding free energy analysis on leading galectin‐3 inhibitors. Synthetic drug molecules GB0139, GB1107, and GB1211 were estimated to have binding free energies of −4.3, −6.7, and −9.5 kcal/mol respectively. This compares to −0.3 and 1.4 kcal/mol for the natural ligands, N‐acetyllactosamine type 1 and type 2, respectively. We calculated the electric fields projected along key bonds in each ligand to further rationalize these results. We find that while the hydroxyl groups of the natural ligands interact reasonably well with residues in galectin‐3's binding pocket, structural dynamics weaken the binding pose and favor interactions with water, sometimes yielding to dissociation. In contrast, the more favorable binding energy of GB1211, leading inhibitor in clinical studies, is associated with strong and constant electric fields across the bonds investigated, suggesting a stiffer binding pose with a stabilizing σ‐hole interaction.

## 1 INTRODUCTION

Galectins are a family of soluble proteins that bind β‐galactose‐containing carbohydrates and participate in cell–cell interactions, cell‐matrix interactions, and transmembrane signaling (Demetriou et al., 2001; Markowska et al., 2011; Nabi et al., 2015). There are 15 known galectins in mammals and 12 known galectin genes in humans (Cummings et al., 2022; Verkerke et al., 2022). Each galectin contains one or two carbohydrate recognition domains (CRDs) that non‐covalently bind β‐galactose (Leffler et al., 2002). Among these, galectin‐3 (Gal‐3) has been extensively researched due to its overexpression in numerous disorders (Sciacchitano et al., 2018), including inflammation (Henderson & Sethi, 2009; Rabinovich & Toscano, 2009; Thiemann & Baum, 2016), cardiovascular diseases (Boer et al., 2009; Lok et al., 2010; Sharma et al., 2004), cancer (Califice et al., 2004; Liu & Rabinovich, 2005; Ruvolo, 2016), and fibrosis (Dang et al., 2012; Henderson et al., 2006; MacKinnon et al., 2012; Nishi et al., 2007). Because of this strong association with various pathologies, Gal‐3 has become a diagnostic biomarker (Dong et al., 2018) and, more recently, a target for therapeutic inhibition (Ahmed et al., 2023; Bouffette et al., 2023). Ideally, synthetic inhibitors would leverage non‐covalent interactions in the CRD to out‐compete natural ligands and suppress functionality to treat disease symptoms.

**FIGURE 1:**
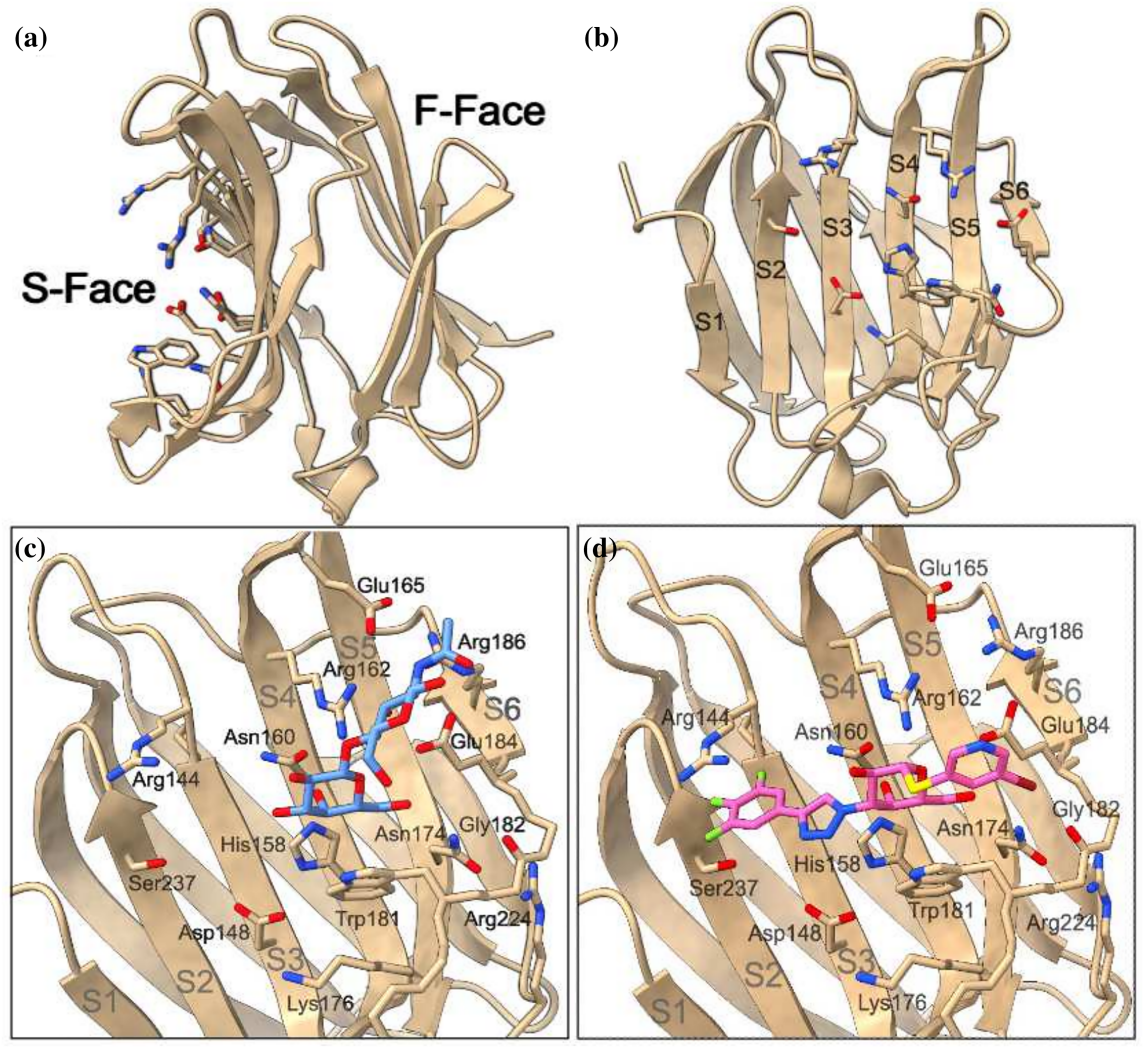
(a) Visualization of the Gal‐3 CRD with the anti‐parallel beta sheets termed the S‐ and F‐faces. Polar residues in the binding pocket on the S face are emphasized. (b) Direct view of the S‐face, with the β strands labeled S1–S6. (c) Binding pose of Gal‐3's natural ligand, LacNAc type 2 (PDB ID: 1KJL). (d) Binding pose of Gal‐3's synthetic inhibitor, GB1211 (PDB ID: 7ZQX). β strands and nearby residues have been labeled. Hydrogens have been omitted for visual clarity.

**FIGURE 2:**
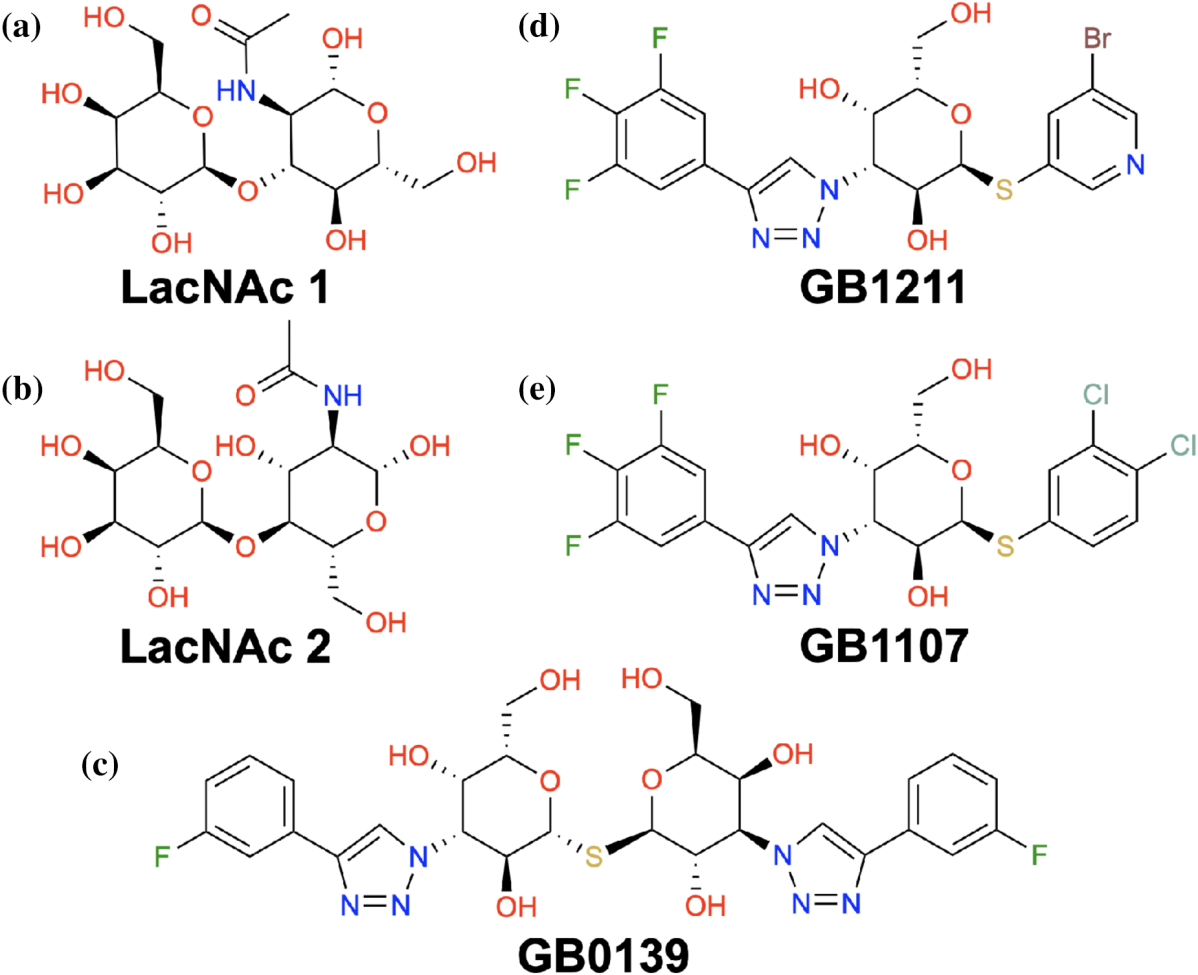
Small‐molecule Gal‐3 ligands and inhibitors: (a) natural ligand LacNAc type 1, (b) synthetic inhibitor GB1211, (c) natural ligand LacNAc type 2, (d) synthetic inhibitor GB1107. Both natural ligands and synthetic inhibitors share the galactose unit.

Gal‐3 is the only chimera‐type galectin with a long flexible N‐terminus tail attached to the highly conserved and globular CRD. The CRD consists of approximately 135 residues that form two antiparallel beta sheets, termed the S‐face and F‐face (Figure 1a). Ligands bind to the slightly concave S‐face, a groove spanning six β‐strands (S1–S6 in Figure 1b) and containing a high density of polar residues (Seetharaman et al., 1998; Leffler et al., 2002). The β‐galactose unit in Gal‐3's natural ligands forms 2–5 hydrogen bonds and exhibits favorable van der Waals interactions with the S4 and S5 β‐strands. Diehl et al. also showed the importance of CH‐π bonding to the tryptophan residue directly below the β‐galactose unit in the binding region, Trp181 (Diehl et al., 2024). Other units, such as glucosamine in N‐acetyllactosamine (LacNAc, see Figure 2a,b), interact with residues of the S6 β‐strand or the solvent (Figure 1c).

**FIGURE 3:**
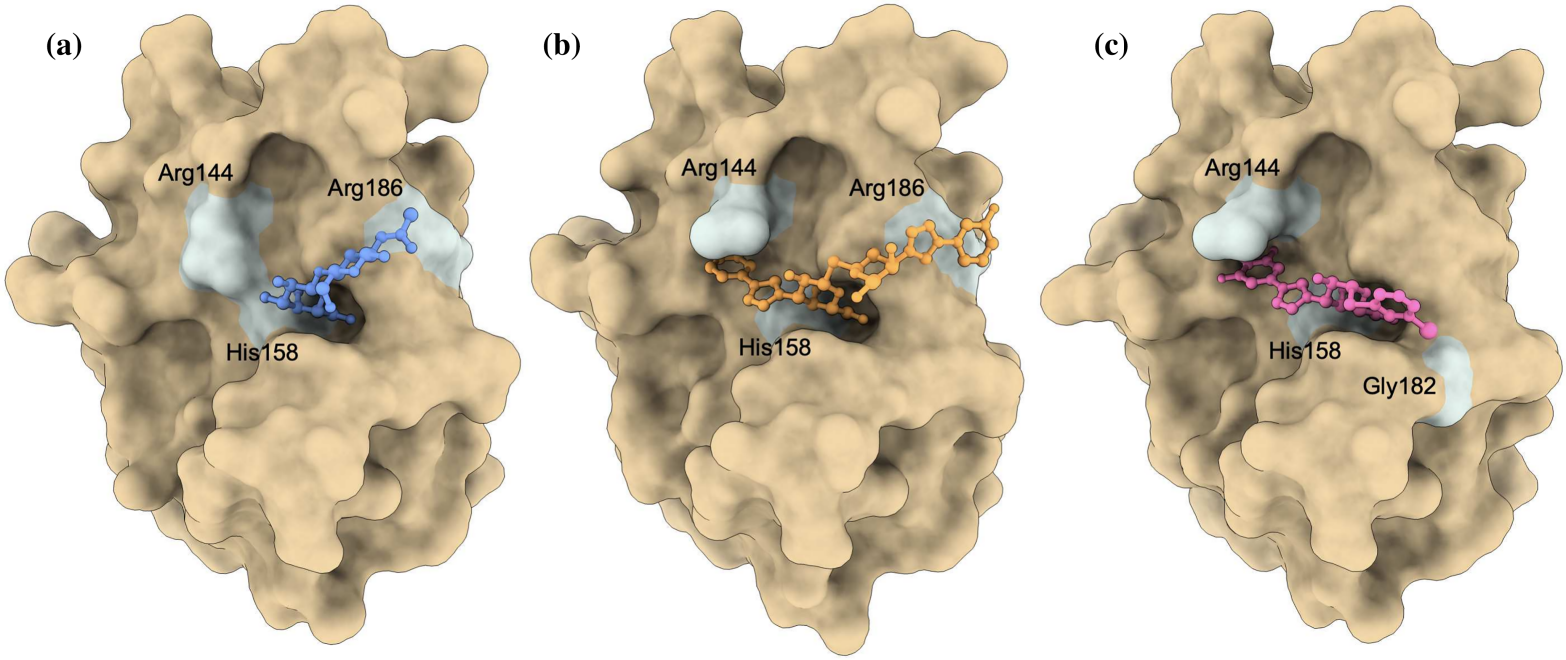
Representation of the Gal‐3 binding pose of (a) disaccharides such as natural ligands LacNAc 1 and 2, (b) thiodigalactosides, such as the first generation synthetic inhibitor GB0139, (c) monogalactopyranosides, such as the second generation synthetic inhibitors GB1211 and GB1107. Generated from PDB crystal structures ID 1KJL, 5H9P, and 7ZQX respectively.

Synthetic Gal‐3 inhibitors have commonly retained the β‐galactose unit while introducing side chains that have more reach to interact with residues on the S2 and S3 β‐strands (Figure 1d). For example, aromatic rings have been added to facilitate interactions with Arg144, a key feature of these synthetic inhibitors (Sörme et al., 2005). One of the early successful Gal‐3 inhibitors was a thio‐disaccharide with substituted aromatic rings, named GB0139 (formerly TD139, see Figure 2c), that showed promise in reducing inflammation and fibrosis in mice models (Delaine et al., 2016; MacKinnon et al., 2012). GB0139 was designed to maintain the established hydrogen bonds of the β‐galactoside unit with His158 and Asn174, while enhancing affinity with Arg144 and Arg186 through two outer aromatic rings. In recent years, a second generation of Gal‐3 inhibitors was established with 1,3‐substituted α‐d‐monogalactopyranosides (Zetterberg et al., 2018, 2022). Affinity for these α anomers is not shared by other galectins, enabling greater Gal‐3 selectivity. Further, 1,3‐substituted α‐d‐monogalactopyranosides were reported to maintain the potent affinity of the previous synthetic ligands such as GB0139 while exhibiting a lower polar surface area, which allowed for oral administration (Zetterberg et al., 2022). Example molecules from this second generation of Gal‐3 inhibitors are GB1211 and GB1107 (Figure 2d,e). GB1211 and GB1107 are compounds 11d and 11b from Galecto Bio, with GB1211 currently in clinical studies (Aslanis et al., 2023). Interestingly, these inhibitors also introduced halogen atoms that are thought to engage in a highly directional σ‐hole bond with Gly182 (Verteramo et al., 2024; Zetterberg et al., 2022). The binding poses of disaccharides, thiodigalactosides and monogalactopyranosides in Gal‐3's CRD, shown in Figure 3, highlights the difference in size and reach between natural and synthetic ligands.

Despite encouraging pharmacokinetics results for GB1211, a clear understanding of how these new compounds bind to Gal‐3's CRD is missing. A detailed molecular picture of Gal‐3's interactions with 1,3‐substituted α‐d‐monogalactopyranosides, that goes beyond the analysis of single structures and crystallographic data, would enable further breakthroughs in the design of potent and selective inhibitors. In this work, we investigate an ensemble of binding configurations of two natural ligands LacNAc type 1 and 2 and the three aforementioned synthetic inhibitors GB0139, GB1211, and GB1107, using molecular dynamics (MD) simulations, binding free energy calculations, and electric field analysis. We use the polarizable AMOEBA force field (Shi et al., 2013) to better account for anisotropic non‐covalent interactions such as CH‐π, cation‐π, and σ‐hole interactions. We find that GB0139 interacts more favorably with Gal‐3 than the natural ligands but still exhibits a relatively strong affinity for water. The second‐generation inhibitors GB1211 and GB1107 bind Gal‐3 even better than GB0139. Interestingly while both GB1211 and GB1107 interact with similar residues, GB1211 has a stiffer binding pose that enables lasting interactions with Gal‐3's CRD and results in an optimized binding energy.

## 2 RESULTS AND DISCUSSION

### 2.1 Natural ligands and synthetic inhibitors in water

**FIGURE 4:**
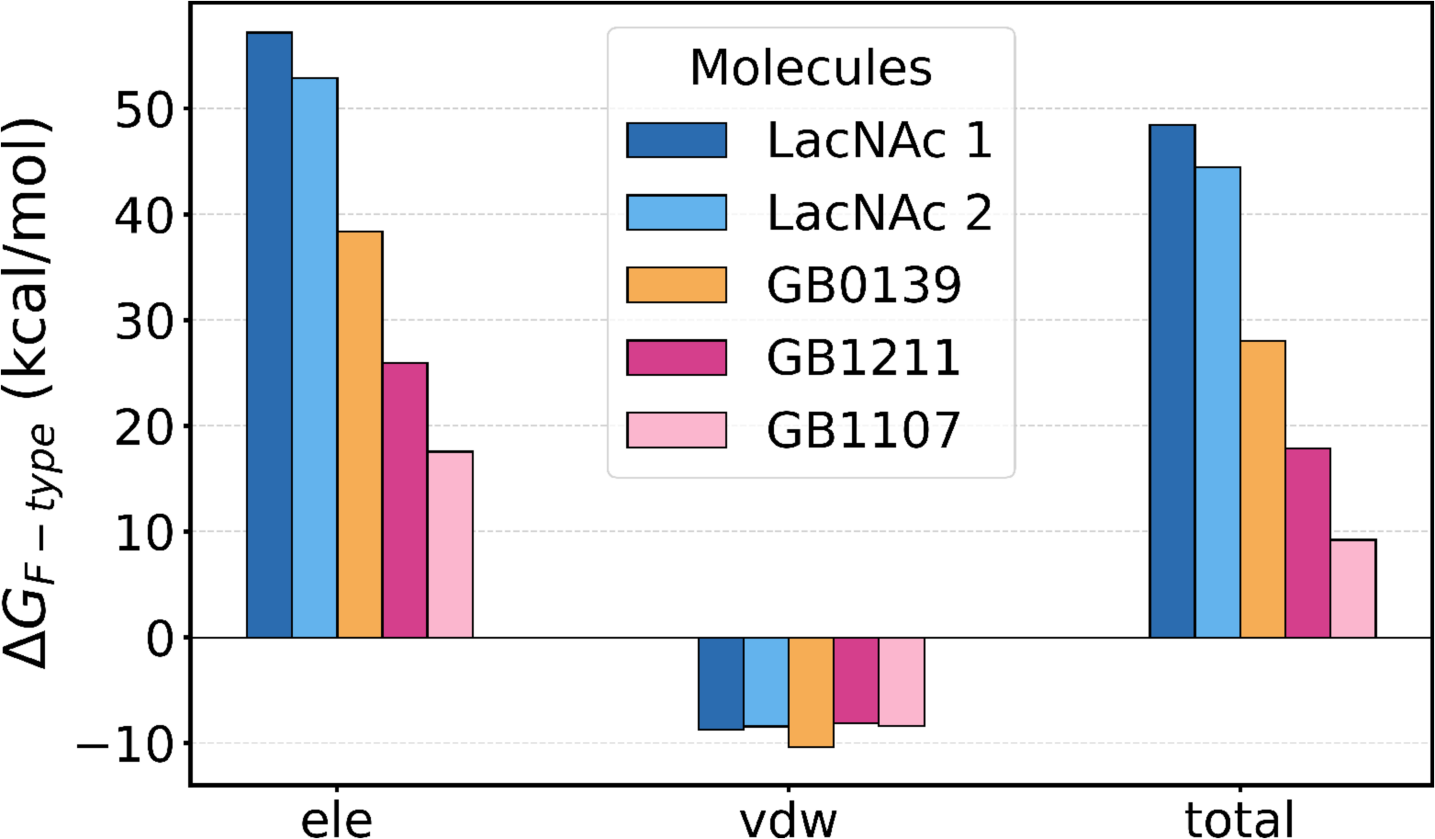
Free energy differences along the path characterizing the free state (see Figure 7) of the ligands LacNAc 1, LacNAc 2, GB0139, GB1211 and GB1107. As described in Methods, the electrostatics (van der Waals) interactions were annihilated (decoupled) over 11 (18) states. The free energy differences between consecutive states were calculated with BAR, summed, and presented as Δ*G*_F‐ele_, Δ*G*_F‐vdw_ and Δ*G*_F‐total_ = Δ*G*_F‐ele_ + Δ*G*_F‐vdw_. The data is also provided in Table S12.

We present in Figure 4 the magnitude of the free energy difference (Δ*G*_F‐total_) associated with the removal of the ligands from water (i.e., the ligand desolvation free energy). The electrostatic (Δ*G*_F‐ele_) and van der Waals (Δ*G*_F‐vdw_) components of Δ*G*_F‐total_ are also shown. Note that, at first approximation, Δ*G*_F‐total_ can be considered as the opposite of the hydration free energy because the ligand intramolecular electrostatics are expected to be negligible. We observe that the natural ligands, LacNAc 1 and 2, have a much greater affinity for water, with a Δ*G*_F‐total_ more than twice that of the second‐generation synthetic inhibitors GB1211 and GB1107. Meanwhile, the first‐generation thio‐disaccharide inhibitor GB0139 proved to be a middle ground between the two groups. We also note that Δ*G*_F‐vdw_ is almost identical for all ligands such that the differences in Δ*G*_F‐total_ are primarily due to variations in Δ*G*_F‐ele_. Interestingly, GB1211 shows almost twice the affinity for water compared to GB1107 (Δ*G*_F‐total_ = 17.9 and 9.2 kcal/mol, respectively). To further rationalize these trends, we computed the electric fields generated by the water molecules onto key bonds in each ligand: the three common hydroxyl bonds (galactose O_2_—H, O_4_—H and O_6_—H), the unique carbon–halogen bonds (C—X), and the central carbon–fluorine bond (C—F).

The electric field projections are presented in Table 1. The electric field experienced by the galactose bonds O_2_—H, O_4_—H, and O_6_—H in LacNAc 1 and 2 ranges from 130 to 165 MV/cm. In this case, the electric field is oriented from O to H such that the electrons are favored to move in the opposite direction, from H to O. This indicates a favorable interaction with water where H is pulled in by the water molecules, mimicking the initiation of a deprotonation event.

**TABLE 1:** Electric fields projected onto specific bonds, in mega volts per centimeter (MV/cm). Values reported are averages over 5 ns MD trajectories of each molecule in water. Positive projections mean that the electric field is oriented from the first atom (O or C) to the second (H, X, or F).

Bond	LacNAc 1	LacNAc 2	GB0139	GB1211	GB1107
O_2_—H	142.2	145.5	141.3	156.5	156.8
O_4_—H	149.4	131.7	140.2	185.5	89.9
O_6_—H	162.9	163.4	150.5	169.8	127.5
C—X	‐	‐	‐	3.5	−4.5
C—F	‐	‐	−14.5	−15.9	−16.0

The electric fields experienced by these hydroxyl bonds in GB0139 are comparable to those of the natural ligands. However, O_2_—H in GB1211 and GB1107 experiences electric fields stronger by about 10 MV/cm than O_2_—H in the other compounds. A similar observation can be made for O_6_—H in GB1211. Further, O_4_—H is subject to a much stronger (weaker) field in GB1211 (GB1107). This partially explains the higher water affinity of GB1211 compared to GB1107. In the case of the C—X bonds, negative electric field projections (i.e., an electric field oriented from X to C) indicate that electrons are favored to move towards the halogen atom. Therefore, the ~−15 MV/cm electric field projections along the C—F bond indicate that the electrons are favored to move outward, towards the F atom, increasing the electronegativity of these functional groups almost identically in GB0139, GB1211, and GB1107. A similar effect is observed for the C—Cl bond in GB1107, although of a lesser magnitude (−4.5 MV/cm). However, the electric field projection is positive along the C—Br bond in GB1211, consistent with the formation of a σ‐hole along this axis. Overall, the electric field data suggests that the substituted aromatic rings in the synthetic ligands disrupt electron distribution along O_4_—H, and to some extent O_6_—H, significantly increasing (decreasing) water affinity of that bond in GB1211 (GB1107). This is largely offset in LacNAc 1, LacNAc 2, and GB0139, by the additional 3–4 hydroxyl bonds of the additional galactose or glucosamine units. Note that the removal of hydroxyl bonds and overall decrease in polar surface area was a design feature in GB1107 and GB1211 to maximize tissue diffusion and delivery compared to GB0139 (Zetterberg et al., 2022).

### 2.2 Natural ligands and synthetic inhibitors in Gal‐3's CRD

**FIGURE 5:**
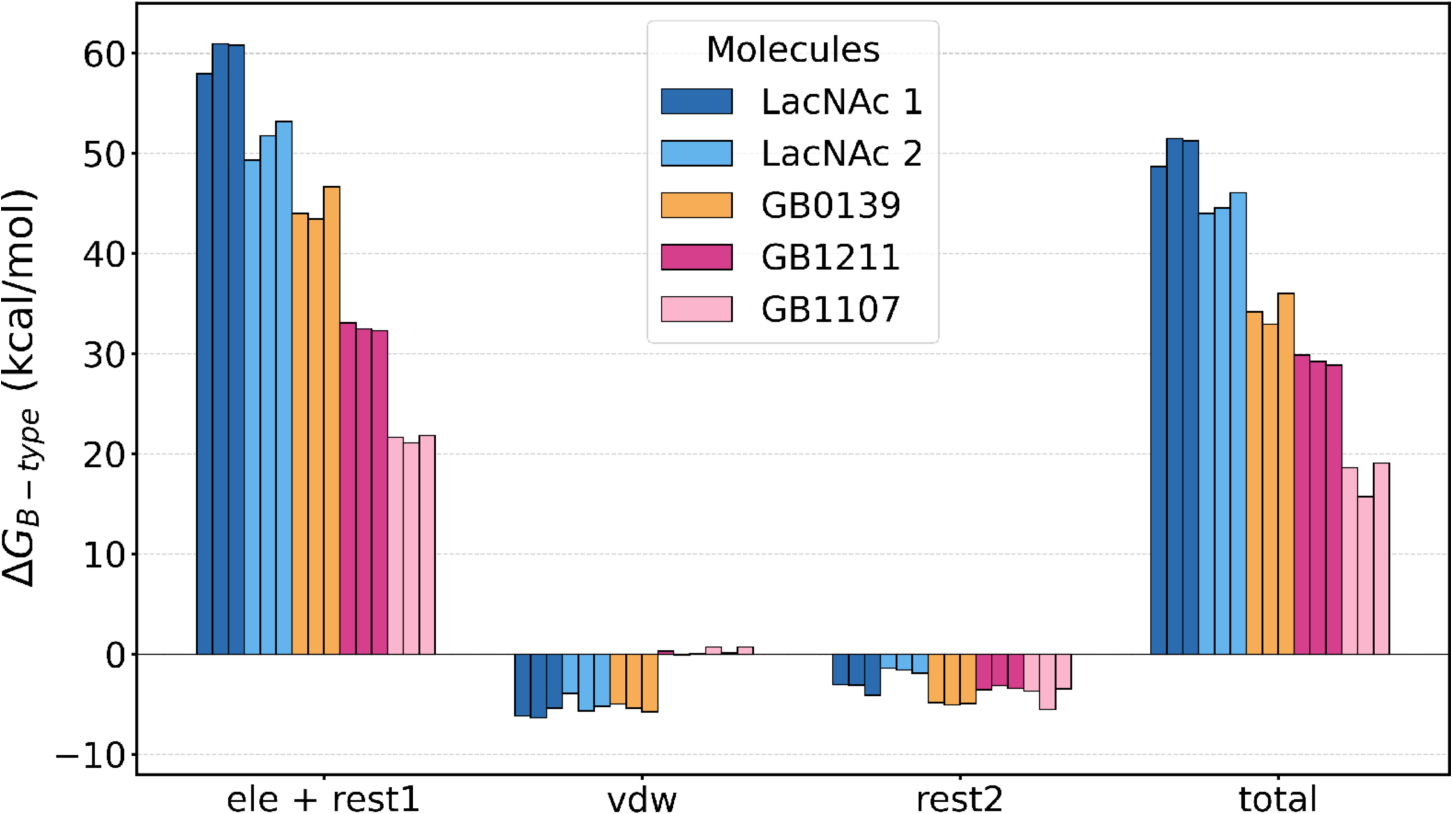
Free energy differences along the path characterizing the bound state (see Figure 7) of the ligands LacNAc 1, LacNAc 2, GB0139, GB1211, and GB1107. As described in Section 4, the electrostatics (van der Waals) interactions were annihilated (decoupled) over 11 (20) states. The free energy differences between consecutive states were calculated with BAR, summed, and presented as Δ*G*_B‐ele+rest‐1_, Δ*G*_B‐vdw_, Δ*G*_B‐rest‐2_, and Δ*G*_B‐total_ = Δ*G*_B‐ele+rest‐1_ + Δ*G*_B‐vdw_ + Δ*G*_rest‐2_. The data is also provided in Table S13.

We present in Figure 5 the magnitude of the free energy difference (Δ*G*_B‐total_) associated with the removal of the ligands from the protein environment. The electrostatic (Δ*G*_B‐ele+rest‐1_), van der Waals (Δ*G*_B‐vdw_), and restraints (Δ*G*_rest‐2_) components of Δ*G*_B‐total_ are also shown. Similarly to the removal of the ligands from water, Δ*G*_B‐total_ is dominated by electrostatics. However, Δ*G*_B‐vdw_ is not constant for all ligands as was previously observed for Δ*G*_F‐vdw_. Rather, LacNAc 1, LacNAc 2, and GB0139 show a similar, negative, Δ*G*_B‐vdw_ while the second‐generation inhibitors, GB1211 and GB1107, exhibit a vanishingly small, positive, Δ*G*_B‐vdw_. This increases the affinity of GB1211 and GB1107 for the protein, albeit to a smaller degree than Δ*G*_B‐ele+rest‐1_. The negative contributions from Δ*G*_rest‐2_ indicate that the release of the multiple distance constraints to a single COM constraint reduces the ligands' affinity for the protein environment. This suggests that the holo (ligand‐bound) and apo (ligand‐absent) forms of Gal‐3 are slightly different, adapting to the substrate. This is especially true for the synthetic inhibitors where additional restraints were used and the magnitude of Δ*G*_rest‐2_ is greater. For LacNAc 2 however, Δ*G*_rest‐2_ is small (1.5–2 kcal/mol), indicating that Gal‐3's CRD does not adapt to the presence of the ligand to the same extent as its synthetic counterparts. Despite this lesser CRD adaptation, LacNAc 1 and 2 have an overall greater Δ*G*_B‐total_, followed by synthetic inhibitors GB1211 and GB1107.

We present in Table 2 the average electric field projections onto the galactose O_2_—H, O_4_—H, and O_6_—H bonds, as well as the projections onto the C—F and unique C—X bonds. In each case, the total field is split into two parts: the contribution from the protein and the contribution from the solvent (water and ions). For example, we observe that the electric field projection onto the O_2_—H bond of all ligands is similar in magnitude to that of the free state and is primarily due to contributions from the solvent. However, even if the magnitude of the electric field along the O_4_—H bond in GB1211 is comparable to that in the free state, it is here dominated by the contribution from the protein. The projection is equally dominated by the protein contribution for the O_4_—H bond in the other compounds, but the total magnitude of the field is greater than it was in the free state. This would suggest that the protein environment is favored to stabilize these hydroxyl groups, especially in GB1107 where the difference between the field in the bound and free states reaches 60 MV/cm, compared to 10–20 MV/cm for the natural ligands and GB0139. In contrast, the electric field projections onto the O_6_—H bond in all ligands but GB0139 are significantly smaller in the bound state than the free state, suggesting that a pure water environment is preferred over the interaction with the CRD for that bond. The decomposition of the protein electric fields into contribution from individual residues (Figures S8–S12) confirms that O_4_—H and O_6_—H engage in hydrogen bonds with His158, Asn174, and Glu184, as reported in the literature (Zetterberg et al., 2018, 2022). Finally, the projections onto the C—F and C—X bonds in GB1211 and GB1107 are overall more negative, by about 5–10 MV/cm, in the bound state compared to the free state. In GB0139, the C—F bond experiences similar or slightly more positive fields in the bound state than in the free state. Overall, the electric field data in the bound state reveal that the protein replaces water sufficiently well to stabilize the hydroxyl groups, which explains why the natural ligands have a more positive Δ*G*_B‐total_ than the synthetic inhibitors. The projections across all bonds in GB1211 are slightly higher than in GB1107 and GB0139, suggesting that the difference between the inhibitors is a combined effect rather than the result of a single interaction.

**TABLE 2:** Average electric fields projected onto specific bonds, in MV/cm, averaged over three replicates of 7 ns for each molecule bound to Gal‐3.

	LacNAc 1	LacNAc 2	GB0139	GB1211	GB1107
O_2_—H prot.	7.7	4.1	−2.6	−0.8	−6.4
O_2_—H sol.	132.4	139.6	157.7	156.9	190.7
O_4_—H prot.	144.0	108.1	171.5	198.7	167.4
O_4_—H sol.	36.6	40.1	−3.5	−10.4	−17.5
O_6_—H prot.	129.2	126.0	165.3	126.6	118.4
O_6_—H sol.	7.1	21.2	9.3	−24.4	−25.1
C—X prot.	‐	‐	‐	3.4	−10.2
C—X sol.	‐	‐	‐	−5.9	−8.1
C—F prot.	‐	‐	−8.7	−21.9	−16.4
C—F sol.	‐	‐	−3.9	2.5	−3.8

*Note*: Positive projections indicate that the electric field is oriented from the first atom (O or C) to the second (H, X, or F). The contributions from each replicate are given in Figures S8–S12. The electric fields for each replicate are given in Tables S14 and S15.

### 2.3 Binding free energies

In this last section, we combine the total free energy difference in the free (Δ*G*_F‐total_) and bound (Δ*G*_B‐total_) states to compute the binding free energy of all five ligands (Δ*G*°) from Equation (2). The results are presented in Table 3. On average, Δ*G*° = −0.3, 1.4, −4.3, −9.5, and −6.7 kcal/mol for LacNAc 1, LacNAc 2, GB0139, GB1211, and GB1107, respectively. We observe that the synthetic inhibitors have a Δ*G*° between 5 and 9 kcal/mol stronger than the natural ligands. GB1211 was also observed to have a greater (>3 kcal/mol) Δ*G*° than GB1107 and GB0139, which aligns with the findings from Zetterberg et al (Zetterberg et al., 2022). Interestingly, the binding energy of GB0139 falls between that of the natural ligands and second‐generation inhibitors, keeping with its shared qualities of both groups.

**TABLE 3:** Binding free energy (Δ*G*°) in kcal/mol for ligand replicates (R1–R3).

Replicates	LacNAc 1			LacNAc 2			GB0139			GB1211			GB1107		
	R1	R2	R3	R1	R2	R3	R1	R2	R3	R1	R2	R3	R1	R2	R3
Δ*G*_F‐total_ in kcal/mol	48.5 ± 0.3			44.5 ± 0.3			28.0 ± 0.4			17.9 ± 0.3			9.2 ± 0.3		
Δ*G*_B‐total_ in kcal/mol	48.7	51.5	51.3	44.0	44.5	46.1	34.2	33.0	36.0	29.9	29.2	28.9	18.6	15.7	19.1
⟨Δ*G*_B‐total_⟩ in kcal/mol	50.5 ± 1.5			44.9 ± 1.1			34.4 ± 1.5			29.3 ± 0.5			17.8 ± 1.8		
Δ*G*_rest‐3_ in kcal/mol	−1.7			−1.8			−2.0			−2.0			−1.9		
Δ*G*° in kcal/mol	1.4	−1.3	−1.1	2.3	1.7	0.2	−4.1	−2.9	−5.9	−10.0	−9.4	−9.0	−7.5	−4.6	−8.0
⟨Δ*G*°⟩ in kcal/mol	−0.3 ± 1.8			1.4 ± 1.4			−4.3 ± 1.9			−9.5 ± 0.8			−6.5 ± 2.1		
Data obtained from binding assays in kcal/mol	−5.50, −7.96^a^ (Hsieh et al., 2015)			−6.11, −8.84^a^ (Hsieh et al., 2015) −7.83 (Salomonsson et al., 2010)/−5.04 (Seetharaman et al., 1998)			−10.71 (Delaine et al., 2016)			−10.36 (Zetterberg et al., 2022)			−10.13 (Zetterberg et al., 2022)		

*Note*: The components Δ*G*_F‐total_, Δ*G*_B‐total_ and Δ*G*_rest‐3_ are also shown (see Equation (2)). ⟨Δ*G*_F‐total_⟩ and ⟨Δ*G*°⟩ are averages over replicas. Experimental binding free energies are converted from dissociation constants.

^a^ Biolayer interferometry at 300 K, fluorescence anisotropy at 277 K.

**FIGURE 6:**
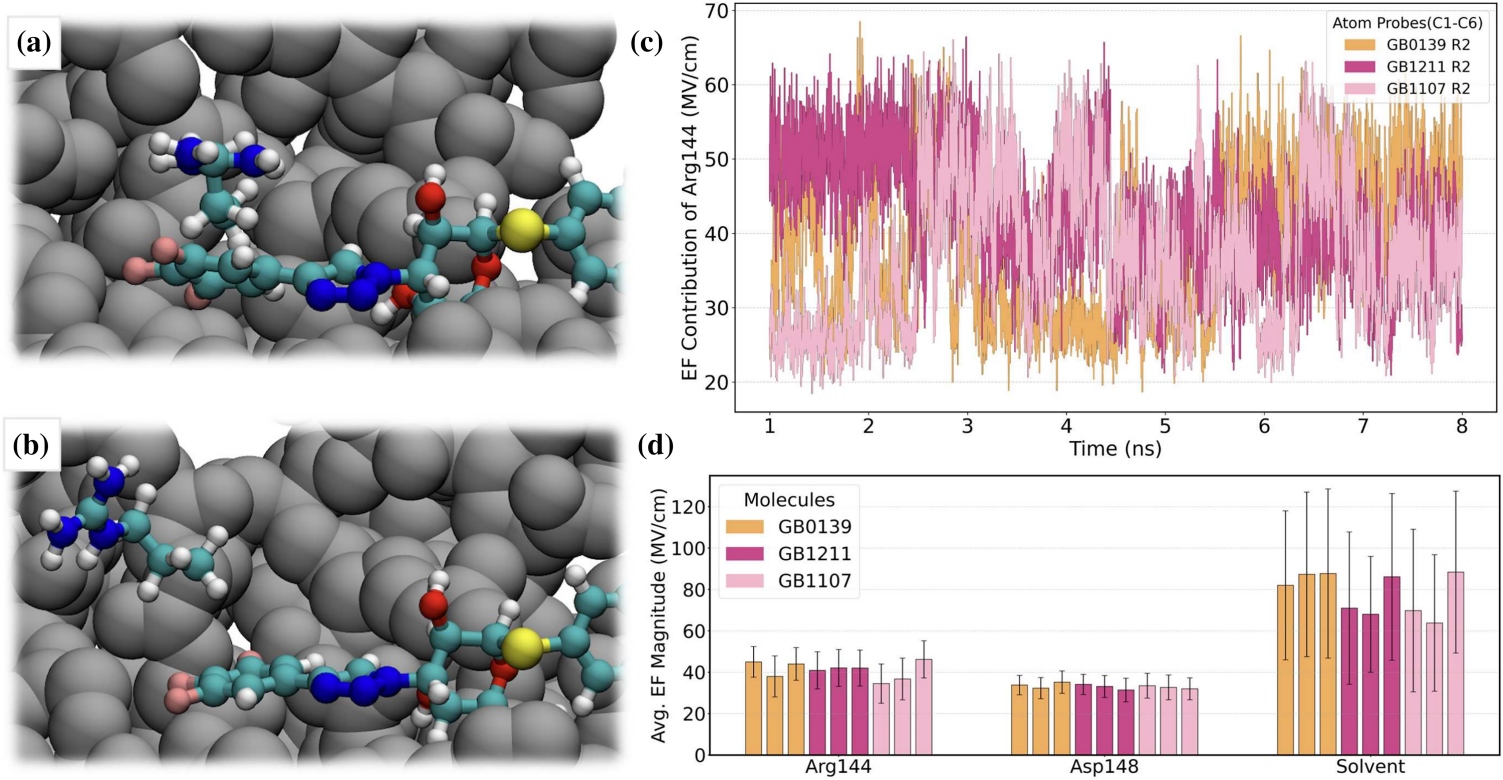
Cation‐π interaction between the fluorinated aromatic ring and Arg144 located in the Gal‐3 CRD. (a) Arg144 is stacked on top of the ring in GB1107. (b) Arg144 has flipped outwards away from GB1107. (c) Time evolution of Arg144's contributed electric field using atomic probes C1–C6 (aromatic carbons) for replicate 2 of all synthetic inhibitors. (d) Arg144, Asp148, and solvent contributions to the magnitude of the electric field, averaged over time and the 6 aromatic carbons.

In addition to the σ‐hole influence of the unique halogen atom in GB1211 and GB1107 described above, a key difference between GB1211 and GB1107 is the cation‐π interactions between the fluorinated ring and Arg144. Indeed, this interaction has been shown to be critical for synthetic Gal‐3 inhibitors, and the addition of an aromatic ring substituent resulted in 5 times greater affinity than the base carbohydrate structure in experimental studies (Sörme et al., 2005). Sörme et al. report that the strength of the cation‐π interaction ranges from −0.4 to −2.4 kcal/mol, and that it does not depend entirely on the electrostatic interactions but also van der Waals and polarization effects. In Figure 6a,b, we show a snapshot representative of Arg144 engaging (disengaging) in (from) the cation‐π interaction with GB1107. To better quantify the strength of this interaction across our various MD simulations, we computed the time evolution of the contribution from Arg144 to the electric field on each of the aromatic carbons in GB0139, GB1211, and GB1107. Note that Arg144 is the top contributor to the total field on these atoms, followed by Asp148. We show in Figure 6c that trajectories where Arg144 is engaging in the cation‐π interaction result in fields around 50 MV/cm, as for GB1211 R2. Meanwhile, the electric field drops to 20 MV/cm when Arg144 is not engaged in the interaction, as it is the case for GB0139 R2 between 3 and 6 ns, GB1107 R2 during the first 2.5 ns, between 4.5 and 6.2 ns and after 7 ns. The data averaged over time and replicates is presented in Figure 6d, as well as the contribution from Asp148 and the solvent, for reference. We see that the contribution from Arg144 is very consistent across all replicates in GB1211, suggesting a sustainable binding pose. Meanwhile, Arg144 is 5–10 MV/cm lower in the second replicate of GB0139 and the first two replicates of GB1107, due to significant amount of time where Arg144 faces away from the aromatic ring, breaking the cation‐π interaction. These results correlate with variations in Δ*G*° across replicates.

Finally, we find that Δ*G*° for the disaccharides (i.e., LacNAc 1, LacNAc 2, and GB0139) was overall more positive than the literature would suggest from converted dissociation constants. Further, our results show LacNAc 1 as the preferential binder, with LacNAc 2 having a positive binding free energy. The trend is contrary to findings from Hsieh et al., who found a binding preference of the Gal‐3 CRD for LacNAc 2 over LacNAc 1 (Hsieh et al., 2015). Hsieh et al. associated the difference in binding between LacNAc 1 and 2 to a different binding pose of the N‐acetylglucosamine unit, as indicated by two dihedral angles characterizing the glycosidic bond in the crystal structure (Hsieh et al., 2015). In Table 4, we show the average and standard deviations of these two angles in our MD simulations, compared with data acquired from crystal structure (Hsieh et al., 2015; Seetharaman et al., 1998).

**TABLE 4:** Characteristic dihedral angles for LacNAc type 1 and 2 across replicates (see Figure S3 for a sketch of these angles). Angle 1 LacNAc 1: O_5_Gal–C_1_Gal–O_3_GlcNAc–C_3_GlcNAc | LacNAc 2: O_5_Gal–C_1_Gal–O_4_GlcNAc–C_4_GlcNAc. Angle 2 LacNAc 1: C_1_Gal–O_3_GlcNAc–C_3_GlcNAc–C_4_GlcNAc | LacNAc 2: C_1_Gal–O_4_GlcNAc–C_4_GlcNAc–C_5_GlcNAc. Data presented as average angle over the sampling MD (standard deviation). The evolution of each angle over time is given in Figures S4–S7.

	Ref (Hsieh et al., 2015; Seetharaman et al., 1998).	R1	R2	R3
Angle 1 (°)				
LacNAc 1	−60	−105.3 (21.7)	−69.0 (9.2)	−69.4 (8.2)
LacNAc 2	−66, −68	−97.1 (20.7)	−70.8 (9.9)	−70.3 (9.8)
Angle 2 (°)				
LacNAc 1	135	101.6 (18.2)	142.7 (7.8)	141.7 (7.6)
LacNAc 2	−108, −103	−137.0 (17.6)	−108.4 (7.5)	−108.5 (7.7)

The first dihedral angle provides the orientation of the β‐galactose unit relative to the glycosidic bond. We observe an average of −70° for LacNAc 1 and 2 in R2 and R3, which is in good agreement with the experimental values. However, R1 for both ligands showed a higher deviation and more negative angle due to larger molecule movements within the binding pocket. The second dihedral angle tracks the orientation of the N‐acetylglucosamine unit relative to the galactose unit. Here again, R1 shows a higher deviation, but R2 and R3 show good agreement with the experimental data. Interestingly, this may explain the 2.5 kcal/mol difference in Δ*G*° observed between R1 and R2/R3 of LacNAc 1 (Table 3). The LacNAc 2 replicates also see a slight decrease in binding free energy compared to R1, however, these results are still more positive than expected from literature.

It is worth noting that the experimental binding energies are not a one‐to‐one comparison with our theoretical estimates, as the fluorescence polarization methods require covalently attaching a fluorophore molecule to the ligand, which was not modeled in the simulations. In fact, Sörme et al. explicitly mentions that fluorescence polarization tends to overestimate the affinity for small carbohydrate‐lectin systems, which would include Gal‐3/LacNAc complexes (Sörme et al., 2004). The conversion from a dissociation constant to a binding affinity can also be a bad comparison when strong protein‐ligand interactions occur outside of the canonical binding location. Indeed, our approach only sampled protein‐ligand configurations close to the bound crystal structures and did not account for alternative binding sites along the groove in the S‐face. Support for the existence of other, potentially more favorable, binding poses is that our first trial for LacNAc 2 resulted in the dissociation of the ligand after 39 ns of simulation time, directly after sampling the first replicate from 31 to 38 ns. Surprisingly, Δ*G*° from this replicate before dissociation was 0.63 kcal/mol. Alternatively, the underestimation of Δ*G*° for LacNAc 1 and 2 could point to a force field issue where AMOEBA overestimates the carbohydrates' affinity for water.

## 3 CONCLUSION

Leading Gal‐3 synthetic inhibitors were studied alongside natural carbohydrate ligands to rationalize binding affinities at the molecular scale. We used the AMOEBA polarizable force field along with alchemical transformations and electric field calculations to understand the ligands' affinity for water and Gal‐3, thereby providing a deeper understanding of the resulting binding energies. We predicted the binding energy of inhibitor GB1211 to be −9.5 kcal/mol on average, while GB1107 exhibited a lower affinity at −6.7 kcal/mol, consistent with experimental data. Electric field calculations suggest that the stronger binding of GB1211 originates from stronger interactions between the protein and the hydroxyl bonds O_4_—H and O_6_—H, as well as a stronger cation‐π interaction with Arg144. However, our estimates for the disaccharides LacNAc 1, LacNAc 2, and GB0139 were much more positive than experimental values, at −0.3, 1.4, and −4.3 kcal/mol, respectively. This may suggest the existence of multiple binding poses for these natural ligands, not sampled in our MD that started from crystal structures. Alternatively, this could also come from a force field issue where AMOEBA overestimates the water affinity of carbohydrates. Further work will involve systematically scaling the van der Waals terms in AMOEBA to assess the later hypothesis and investigating the interaction profile of these ligands in other galectin members. Indeed, the relative difference between the electric field projections and binding energies of LacNAc 1, LacNAc 2, GB0139, GB1211, and GB1107 complexed in Gal‐3 and other Gal forms will further assess the validity of our methods and provide a molecular picture of selectivity.

## 4 METHODS

### 4.1 System preparation

The crystal structures PDB ID 4XBN (Hsieh et al., 2015), 1KJL (Sörme et al., 2005), 5H9P (Hsieh et al., 2016), 7ZQX (Zetterberg et al., 2022), 6EOL (Zetterberg et al., 2018) were used to prepare the protein‐ligand systems for LacNAc 1, LacNAc 2, GB0139, GB1211, and GB1107, respectively. The protein files were cleaned of crystallography artifacts and any missing atoms from terminal ends were added to result in 138 residues comprising the Gal‐3 CRD but omitting the flexible N‐terminus tail. Protons were added using the reduce program (Word et al., 1999). The histidines were consistently protonated on the *ε* nitrogen (HIE) with the exception of His158, present at the binding site, which was protonated on the *δ* nitrogen (HID) based on previous structural studies (Hsieh et al., 2015; Sörme et al., 2005), and NMR spectroscopy data (Manzoni et al., 2018). The tinker8 software package (Rackers et al., 2018) was used to solvate the protein–ligand systems in a 60^3^ Å^3^ cubic water box, and the +4 total charge of the protein was balanced with 10 Na^+^ ions and 14 Cl^−^ ions. For the simulations of the ligand in water, the molecules were placed in a 30^3^ Å^3^ cubic box without ions.

### 4.2 Molecular dynamics

poltype2 (Walker et al., 2022) was used to generate the AMOEBA parameters for the ligand molecules, while the amoebabio18 parameters (Shi et al., 2013; Zhang et al., 2018) were used for the protein, water, and ions. GPU‐enabled tinker9 (Rackers et al., 2018; Wang, 2021) was used to minimize the energy of each system and run the MD simulations. The bound and free systems were run at 300 K in the NPT ensemble with the Nosé‐Hoover thermostat and barostat for an initial 30 ns equilibration period. The final atomic coordinates and velocities were used as inputs for the first round of thermodynamic state simulations, which we call replicate 1 (R1). The bound systems were further equilibrated to start a second replicate (R2) at 50 ns, then a third (R3) at 70 ns. Root‐mean‐square deviation (RMSD) and root‐mean‐square fluctuation (RMSF) plots are shown in Figures S1 and S2, respectively.

### 4.3 Binding free energy calculations

**FIGURE 7:**
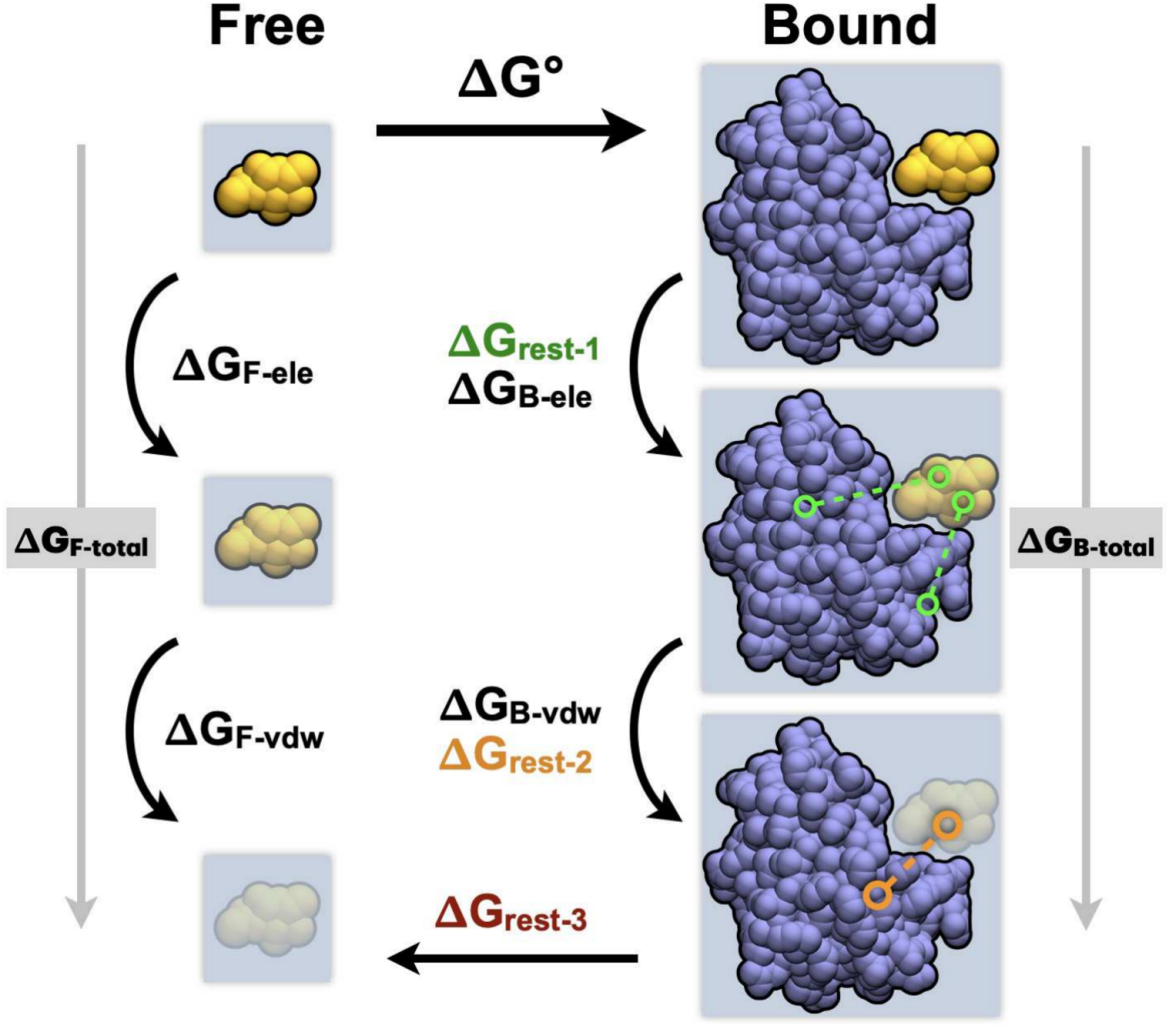
Absolute alchemical thermodynamic cycle showing the paths through the ‘Free’ (i.e., in water) and ‘Bound’ (i.e., in Gal‐3) ligand states to compute the binding free energy Δ*G*°. In each path, the electrostatic and van der Waals interactions are annihilated and decoupled, respectively.

We calculate the binding free energy (Δ*G*°) of Gal‐3‐ligand complexes according to the thermodynamic cycle presented in Figure 7. For each complex, the ligand is modeled in its free (i.e., in water) and bound (i.e., with Gal‐3) states through multiple stages. First, the electrostatic interactions of the ligand are annihilated, meaning both intramolecular and intermolecular interactions are scaled off. Then, the van der Waals interactions between the ligand and its environment are decoupled, meaning the intermolecular van der Waals forces are scaled off while the intramolecular ones remain active. This scaling off of both electrostatics and van der Waals interactions results in a fully decoupled ligand that has effectively been moved to the gas phase. In this work, we repeat this process in both water and the protein environment. To prevent ligand dissociation during the stages where the electrostatics and van der Waals interactions are turned down, we use restraints to maintain its position and orientation in the binding pocket, as detailed below.

#### 4.3.1 Restraints

In this work, we implement multiple distance restraints to sample the relevant configurational space through flat‐bottomed harmonic potentials, defined as:

$$V_{\text{flat}}\left(d\right)=\left\{\begin{array}{ll} k_{\text{flat}}{\left(d-r_{i}\right)}^{2} & \text{if}\;d<r_{i}\\ 0 & \text{if}\;r_{i}\leq d\leq r_{o}\\ k_{\text{flat}}{\left(d-r_{o}\right)}^{2} & \text{if}\;d>r_{o} \end{array}\right.,$$

where *k*_flat_ is the force constant (kcal/mol), *r*_i_ and *r*_o_ the inner and outer radius in which the distance *d* is allowed to freely fluctuate. The radii were chosen to be around 3 standard deviations from the average value, with the intention to be non‐invasive to the natural ligand interactions. The anchor residues were checked for low RMSF values and the anchor atoms were chosen to be heavy atoms (carbon, oxygen, or nitrogen). Specific details of atom indexes on which restraints were applied, *r*_i_, *r*_o_, and *k*_flat_ are given in Tables S1–S3. In brief, the hydrogen bonds from the galactose unit for all ligands were mimicked by restraining the Gal‐O_4_ and Gal‐O_6_ atoms to nitrogen atoms in His158 and Asn174, respectively. The LacNAc molecules were additionally tethered by the anomeric O atom to the nitrogen in Arg162 to keep the carbohydrate against the protein without restraining the rotational freedom of the glucosamine unit along the glycosidic bond. Since the synthetic ligands are longer molecules, restraints were instead placed at both ends. The shared fluorinated aromatic ring was tethered to the backbone nitrogen of Gly238. For GB1107 and GB1211, one restraint mimicked the halogen bond between the chlorine or bromine atom and the carbonyl oxygen of Gly182. For GB0139, a restraint was placed to mimic the hydrogen bond from O_2_ on the second galactose unit to Glu184. Due to the length of this inhibitor, a fifth restraint was added between the second aromatic ring and Arg186. The restraints were intended to mimic natural interactions and be non‐invasive to the unbiased MD simulations.

To decouple the degrees of freedom between protein and ligand, we use a harmonic restraint, as implemented in tinker9 (Rackers et al., 2018; Wang, 2021), between the center of mass of the ligand and protein binding region. The harmonic restraint potential is defined as:

$$V_{\text{harm}}\left(d\right)=k_{\text{harm}}{\left(d-d_{0}\right)}^{2}$$

where *k*_harm_ is the force constant (*k*_harm_ = 15 kcal/mol here), *d*_0_ the equilibrium distance between selections, and *d* the current distance. The protein atom center of mass (COM) selection utilized heavy atoms within about 8–10 Å of the ligand COM, and the atom indices are listed in Table S4.

#### 4.3.2 Thermodynamic window settings

The scaling off of the electrostatic and van der Waals interactions is achieved over multiple parallel MD simulations, also referred to as thermodynamic states, using a scaling parameter (*λ*). *λ* is incrementally decreased from 1 (fully interacting ligand) to 0 (non‐interacting ligand) over *k* steps.

For the free ligand in water, we used 29 total thermodynamic states (Table S5). States 1–11 were used to scale off the ligand's electrostatic interactions (*λ*_ele_ = 1.0, 0.9, 0.8, 0.7, 0.6, 0.5, 0.4, 0.3, 0.2, 0.1, 0.0) and states 12–29 were used to scale off the van der Waals forces (*λ*_vdw_ = 0.975, 0.95, 0.9, 0.85, 0.8, 0.75, 0.7, 0.65, 0.6, 0.55, 0.5, 0.4, 0.3, 0.2, 0.1, 0.05, 0.025, 0.0). For the protein‐bound ligand, we used 34 total thermodynamic states (Table S6). States 1–11 were used to scale off the electrostatic interactions (*λ*_ele_ = 1.0, 0.9, 0.8, 0.7, 0.6, 0.5, 0.4, 0.3, 0.2, 0.1, 0.0) and states 12–31 were used to scale off the van der Waals forces (*λ*_vdw_ = 0.975, 0.95, 0.9, 0.85, 0.8, 0.75, 0.725, 0.7, 0.65, 0.625, 0.6, 0.55, 0.5, 0.4, 0.3, 0.2, 0.1, 0.05, 0.025, 0.0). The flat‐bottom harmonic protein–ligand restraints were activated linearly over the first five states (*k*_flat_ = 0, 10, 20, 30, 40 kcal/mol) and were kept at *k*_flat_ = 40 kcal/mol for the remainder of the steps. Once the ligand was fully decoupled (*λ*_ele_ = 0.0, λ_vdw_ = 0.0) states 32–33 were used to scale down the flat‐bottom restraint strength (*k*_flat_ = 20.5 kcal/mol). In step 34, the multiple distance restraints were replaced with a single harmonic restraint (*k*_harm_ = 15 kcal/mol) between the COM of the ligand and the COM of a selection of protein atoms in the binding site. See also Table S6 for tabulated values of lambda_ele_, lambda_vdw_, *k*_flat_, and *k*_harm_ for each thermodynamic state.

Each *λ* thermodynamic state was run in parallel with the same initial coordinates and velocities. The free states were simulated for 6 ns total, 1 ns equilibration, and 5 ns sampling. The bound states were simulated for 8 ns total, 1 ns equilibration, and 7 ns sampling. The coordinates were printed every 1 ps for both systems, resulting in 5000 samples for each of the free states and 7000 samples for the bound states.

### 4.4 Computing free energy differences

The incremental change between successive *λ* allows for better phase‐space overlap between neighboring thermodynamic states. This is necessary for the convergence of free energy estimators, such as the Bennett acceptance ratio (BAR) (Bennett, 1976; Ytreberg et al., 2006) used in this work. BAR estimates the free energy difference of each set of neighboring states using their respective Boltzmann factors. Here, we use the tinker9 (Rackers et al., 2018; Wang, 2021) implementation of BAR where the statistical error is estimated through bootstrapping (error listed in Tables S7–S11 for each ligand). Considering the free energy difference, $\Delta G_{\lambda_{k}\lambda_{k+1}}$, between the two successive *λ*_*k*_ and *λ*_*k*+1_ states (*k* is the step number), it yields:

(1) $$\begin{array}{l} \sum_{q_{k}=1}^{n_{\lambda_{k}}}\frac{1}{1+\exp\left(\ln\left(\frac{n_{\lambda_{k}}}{n_{\lambda_{k+1}}}\right)+\beta \Delta U_{\lambda_{k}\lambda_{k+1}}-\beta \Delta G_{\lambda_{k}\lambda_{k+1}}\right)}\\ \;=\sum_{q_{k+1}=1}^{n_{\lambda_{k+1}}}\frac{1}{1+\exp\left(\ln\left(\frac{n_{\lambda_{k+1}}}{n_{\lambda_{k}}}\right)-\beta \Delta U_{\lambda_{k}\lambda_{k+1}}+\beta \Delta G_{\lambda_{k}\lambda_{k+1}}\right)}, \end{array}$$

where $\beta =\frac{1}{k_{B}T}$, *k*_B_ the Boltzmann constant and *T* the temperature. $n_{\lambda_{k}}$ and $n_{\lambda_{k+1}}$ are the number of samples (i.e. MD simulation frames) from states *λ*_*k*_ and *λ*_*k*+1_, respectively. $\Delta U_{\lambda_{k}\lambda_{k+1}}=U_{\lambda_{k+1}}\left(q\right)-U_{\lambda_{k}}\left(q\right)$, where $U_{i}\left(q\right)$ is the potential energy of frame *q* in state *i*. $\Delta G_{\lambda_{k}\lambda_{k+1}}$ must be numerically solved. In practice, BAR takes each MD frame from states *λ*_*k*_ and *λ*_*k*+1_, calculates their energy with each of the states' potential energy functions, and iteratively solves for $\Delta G_{\lambda_{k}\lambda_{k+1}}$ using Equation (1).

The energy difference between two stages of the thermodynamic cycle described in Figure 7 is computed by summing the $\Delta G_{\lambda_{k}\lambda_{k+1}}$ over the number of steps *k* required to turn on/off the electrostatic or van der Waals interactions. The detailed $\Delta G_{\lambda_{k}\lambda_{k+1}}$ for each segment of the thermodynamic cycle is given in Tables S7–S11 for each replicate.

Overall, we define the binding free energy (Δ*G*°) as:

(2) $$\Delta G^{{}^{\circ}}=\begin{array}{l} \underset{\Delta G_{F‐\text{total}}}{\underbrace{\Delta G_{F‐\mathrm{ele}}+\Delta G_{F‐\mathrm{vdw}}}}\\ -\left(\underset{\Delta G_{B‐\text{total}}}{\underbrace{\Delta G_{B‐\mathrm{ele}}+\Delta G_{\text{rest}‐1}+\Delta G_{B‐\mathrm{vdw}}+\Delta G_{\text{rest}‐2}}}\right)\\ -\;\Delta G_{\text{rest}‐3}, \end{array}$$

where Δ*G*_F‐ele_ is the free energy difference associated with the annihilation of the electrostatic interactions of the ligand in water (i.e., “free” state), Δ*G*_F‐vdw_ the free energy difference associated with the decoupling of the van der Waals forces of the ligand in water, Δ*G*_B‐ele_ the free energy difference associated with the annihilation of the electrostatic interactions of the ligand in the protein environment (i.e., bound state), Δ*G*_B‐vdw_ the free energy difference associated with the decoupling of the van der Waals forces of the ligand in the protein environment, Δ*G*_rest‐1_ is the free energy difference associated with turning on multiple distance restraints to maintain the ligand in the binding pocket and Δ*G*_rest‐2_ is the free energy difference associated with collapsing the multiple distance restraints to a single distance restraint. All of these free energy differences are computed through the BAR method. Finally, Δ*G*_rest‐3_ is the free energy associated with releasing the non‐interacting ligand from the binding pocket to bulk solvent (Hermans & Wang, 1997). This term is a correction due to sampling and convergence issues of letting the ligand explore the entire simulation box and is defined as:

(3) $$\Delta G_{\text{rest}‐3}=-k_{B}T\ln\left(C^{0}V_{\text{restraint}}\right),$$

where *V*_restraint_ is the volume integral of the restrained ligand and *C* ^0^ the standard concentration (1 mol/L or 1 mol/1660 Å^3^). Here, we use the tinker8 freefix (Rackers et al., 2018) utility to compute Δ*G*_rest‐3_, closing the alchemical cycle in Figure 7.

### 4.5 Electric field calculations

We use ELECTRIC (Nash et al., 2020) to calculate the electric field for each ligand replicate from the AMOEBA MD sampling simulations (6 ns for the free ligands and 8 ns for the protein‐bound molecules, all fully interacting).

The electric field projection along a bond *ij* between atoms *i* and *j* was calculated as: $E_{\text{proj}}^{\mathrm{ij}}=\frac{{\vec{E}}^{i}+{\vec{E}}^{j}}{2}\cdot {\vec{u}}^{\mathrm{ij}}$ where $E_{\text{proj}}^{\mathrm{ij}}$ is the projected electric field, ${\vec{E}}^{i}$ the electric field at atom *i*, ${\vec{E}}^{j}$ the electric field at atom *j*, and ${\vec{u}}^{\mathrm{ij}}$ is the unit bond axis between *i* and *j*. For the natural ligands (LacNAc 1 and 2), the projected electric field was analyzed along the O_2_—H_2_, O_4_—H_4_, and O_6_—H_6_ bonds in the galactose unit. This selection was repeated for synthetic inhibitors, with additional projections along the C—X bond (X = bromine or chlorine) and the central C—F bond. For the fluorinated aromatic ring, the six carbons were used as electric field probes. Instead of projections, the magnitude of the electric field was computed.

## AUTHOR CONTRIBUTIONS

**Luke Newman:** Writing – original draft; investigation; methodology; validation; visualization; data curation. **Valerie Vaissier Welborn:** Funding acquisition; writing – original draft; writing – review and editing; formal analysis; supervision; conceptualization.

## FUNDING INFORMATION

GlycoMIP, National Science Foundation Materials Innovation Platform, Funded through Cooperative Agreement DMR‐1933525.

## CONFLICT OF INTEREST STATEMENT

There are no conflicts to declare.

## Supporting information


**TABLE S1:** Flat‐bottom harmonic restraint settings for LacNAc 1 and 2, including the atom index, inner (*r*_i_), outer (*r*_o_) radii and force constant (*k*_flat_) when not being scaled down.
**FIGURE S1:** Root mean square deviation (RMSD) of the protein atoms as a function of simulation time.
**TABLE S2:** Flat‐bottom harmonic restraint settings for GB0139, including the atom index, inner (*r*_i_), outer (*r*_o_) radii and force constant (*k*_flat_) when not being scaled down.
**TABLE S3:** Flat‐bottom harmonic restraint settings for GB1211 and GB1107, including the atom index, inner (*r*_i_), outer (*r*_o_) radii and force constant (*k*_flat_) when not being scaled down.
**FIGURE S2:** Root mean square fluctuation (RMSF) of (a) LacNAc 1, (b) LacNAc 2, (c) GB0139, (d) GB1211, and (e) GB1107 averaged over the 78 ns simulation time. The anchor atoms used in the flat‐bottom harmonic restraints are denoted with black markers.
**TABLE S4:** Harmonic restraint setting between the center of mass of each ligand and the binding pocket, with a distance (*r*) and a force constant (*k*_harm_). The protein selection for the restraint included the following atom indices: 496–500, 719–723, 752, 755–758, 783–789, 836, 841–845, 961, 962, 967–969, 994–997, 1097–1099, 1103–1112, 1124, 1153, 1158–1162, 1190–1194.
**TABLE S5:** Enumeration of all the thermodynamic states simulated for the ligands in free state (i.e., in water). The scaling factor to turn down electrostatic (*λ*ele) and van der Waals (*λ*vdw) are given in each case.
**TABLE S6:** Enumeration of all the thermodynamic states simulated for the ligands in bound state (i.e., in Gal‐3). The scaling factor to turn down electrostatic (*λ*ele) and van der Waals (*λ*vdw), as well as the force constant for the restraints, are given in each case.
**TABLE S7:** BAR calculation of the free energy difference between each thermodynamic states for free and bound LacNAc 1 (three replicates R1–R3). The bootstrapping error is also given in each case.
**TABLE S8:** BAR calculation of the free energy difference between each thermodynamic states for free and bound LacNAc 2 (three replicates R1–R3). The bootstrapping error is also given in each case.
**TABLE S9:** BAR calculation of the free energy difference between each thermodynamic states for free and bound GB1211 (three replicates R1–R3). The bootstrapping error is also given in each case.
**TABLE S10:** BAR calculation of the free energy difference between each thermodynamic states for free and bound GB1107 (three replicates R1–R3). The bootstrapping error is also given in each case.
**TABLE S11:** BAR calculation of the free energy difference between each thermodynamic states for free and bound GB0139 (three replicates R1–R3). The bootstrapping error is also given in each case.
**FIGURE S3:** Representation of the two glycosidic angles (highlighted with magenta and green lines) used to describe binding pose of (a) LacNAc type 1 and (b) LacNAc type 2.
**FIGURE S4:** Dihedral angle 1 for LacNAc 1 over time, showing the variations between replicates.
**FIGURE S5:** Dihedral angle 2 for LacNAc 1 over time, showing the variations between replicates.
**FIGURE S6:** Dihedral angle 1 for LacNAc 2 over time, showing the variations between replicates.
**FIGURE S7:** Dihedral angle 2 for LacNAc 2 over time, showing the variations between replicates.
**FIGURE S8:** Electric field contributions for LacNAc type 1 projected along the (a) O_4_—H bond and (b) O_6_—H bond. Residues 113–250 comprise the protein amino acids, residue 251 is the ligand contribution, and the solvent is comprised of water, sodium, and chlorine which are residues 252, 253, and 354 respectively.
**FIGURE S9:** Electric field contributions for LacNAc type 2 projected along the (a) O_4_—H bond and (b) O_6_—H bond. Residues 113–250 comprise the protein amino acids, residue 251 is the ligand contribution, and the solvent is comprised of water, sodium, and chlorine which are residues 252, 253, and 354 respectively.
**FIGURE S10:** Electric field contributions for GB0139 projected along the (a) O_4_—H bond and (b) O_6_—H bond. Residues 113–250 comprise the protein amino acids, residue 251 is the ligand contribution, and the solvent is comprised of water, sodium, and chlorine which are residues 252, 253, and 354 respectively.
**FIGURE S11:** Electric field contributions for GB1107 projected along the (a) O_4_—H bond and (b) O_6_—H bond. Residues 113–250 comprise the protein amino acids, residue 251 is the ligand contribution, and the solvent is comprised of water, sodium, and chlorine which are residues 252, 253, and 354 respectively.
**FIGURE S12:** Electric field contributions for GB1107 projected along the (a) O_4_—H bond and (b) O_6_—H bond. Residues 113–250 comprise the protein amino acids, residue 251 is the ligand contribution, and the solvent is comprised of water, sodium, and chlorine which are residues 252, 253, and 354 respectively.
**TABLE S12:** Free energy differences between thermodynamic states that ‘disappear’ the ligand from water. As described in Methods, the electrostatics (van der Waals) interactions were annihilated over 10 (18) states. The free energy differences between consecutive states were calculated with BAR, summed, and presented as Δ*G*F‐ele and Δ*G*F‐vdw. The total Δ*G*F‐total = Δ*G*F‐ele + Δ*G*F‐vdw.
**TABLE S13:** Free energy differences between thermodynamic states that ‘disappear’ the ligand from the protein. As described in Methods, the electrostatics (van der Waals) interactions were annihilated over 10 (20) states. The free energy differences between consecutive states were calculated with BAR, summed, and presented as Δ*G*B‐ele and Δ*G*B‐vdw. The total Δ*G*B‐total = Δ*G*B‐ele + Δ*G*B‐vdw + Δ*G*rest‐2.
**TABLE S14:** Electric fields projected onto specific bonds in LacNAc 1, LacNAc 2 and GB0139, in MV/cm. Values reported are averages over 7 ns MD trajectories of each molecule bound to Gal‐3, for each replicate. Positive projections mean that the electric field is oriented from the first atom (O or C) to the second (H, X, or F).
**TABLE S15:** Electric fields projected onto specific bonds in GB1211 and GB1107, in MV/cm. Values reported are averages over 7 ns MD trajectories of each molecule bound to Gal‐3, for each replicate. Positive projections mean that the electric field is oriented from the first atom (O or C) to the second (H, X, or F).

## Data Availability

The data supporting this article have been included as part of Appendix S1. Input files, including Poltype 2 and Tinker 9 input with parameter files, are available at https://github.com/WelbornGroup/.
