# Supplementary material for: Binding free energy analysis of galectin‐3 natural ligands and synthetic inhibitors
Source: Protein Sci. 2025 May 22;34(6):e70143. doi: 10.1002/pro.70143 (PMC12096018; doi:10.1002/pro.70143)
Supplement: Supplementary file 1 — TABLE S1: Flat‐bottom harmonic restraint settings for LacNAc 1 and 2, including the atom index, inner (r i), outer (r o) radii and force constant (k flat) when not being scaled down. FIGURE S1: Root mean square deviation (RMSD) of the protein atoms as a function of simulation time. TABLE S2: Flat‐bottom harmonic restraint settings for GB0139, including the atom index, inner (r i), outer (r o) radii and force constant (k flat) when not being scaled down. TABLE S3: Flat‐bottom harmonic restraint settings for GB1211 and GB1107, including the atom index, inner (r i), outer (r o) radii and force constant (k flat) when not being scaled down. FIGURE S2: Root mean square fluctuation (RMSF) of (a) LacNAc 1, (b) LacNAc 2, (c) GB0139, (d) GB1211, and (e) GB1107 averaged over the 78 ns simulation time. The anchor atoms used in the flat‐bottom harmonic restraints are denoted with black markers. TABLE S4: Harmonic restraint setting between the center of mass of each ligand and the binding pocket, with a distance (r) and a force constant (k harm). The protein selection for the restraint included the following atom indices: 496–500, 719–723, 752, 755–758, 783–789, 836, 841–845, 961, 962, 967–969, 994–997, 1097–1099, 1103–1112, 1124, 1153, 1158–1162, 1190–1194. TABLE S5: Enumeration of all the thermodynamic states simulated for the ligands in free state (i.e., in water). The scaling factor to turn down electrostatic (λele) and van der Waals (λvdw) are given in each case. TABLE S6: Enumeration of all the thermodynamic states simulated for the ligands in bound state (i.e., in Gal‐3). The scaling factor to turn down electrostatic (λele) and van der Waals (λvdw), as well as the force constant for the restraints, are given in each case. TABLE S7: BAR calculation of the free energy difference between each thermodynamic states for free and bound LacNAc 1 (three replicates R1–R3). The bootstrapping error is also given in each case. TABLE S8: BAR calculation of the free energy difference bet [file PRO-34-e70143-s001.pdf]

Binding Free Energy Analysis of Galectin-3 Natural Ligands and Synthetic Inhibitors Supporting Information

Luke A. Newman† and Valerie Vaissier Welborn*,†,‡

†*Department of Chemistry, Virginia Tech, Blacksburg, VA 24060, USA*

‡*Macromolecules Innovation Institute (MII), Virginia Tech, Blacksburg, VA 24060, USA*

E-mail: lnewman2@vt.edu,vwelborn@vt.edu

Table S1: Flat-bottom harmonic restraint settings for LacNAc 1 and 2, including the atom index, inner (r_i), outer (r_o) radii and force constant (k_{flat}) when not being scaled down.

	LacNAc 1		
	Restraint 1	Restraint 2	Restraint 3
Atom Index	2240, 997	2237, 723	2234, 789
r_i, r_o in Å	2.70, 3.60	2.70, 3.45	3.10, 4.40
k_{flat} in kcal/mol	40	40	40

	LacNAc 2		
	Restraint 1	Restraint 2	Restraint 3
Atom Index	2240, 997	2237, 723	2231, 789
r_i, r_o in Å	2.50, 3.9	2.55, 4.50	2.65, 6.50
k_{flat} in kcal/mol	40	40	40

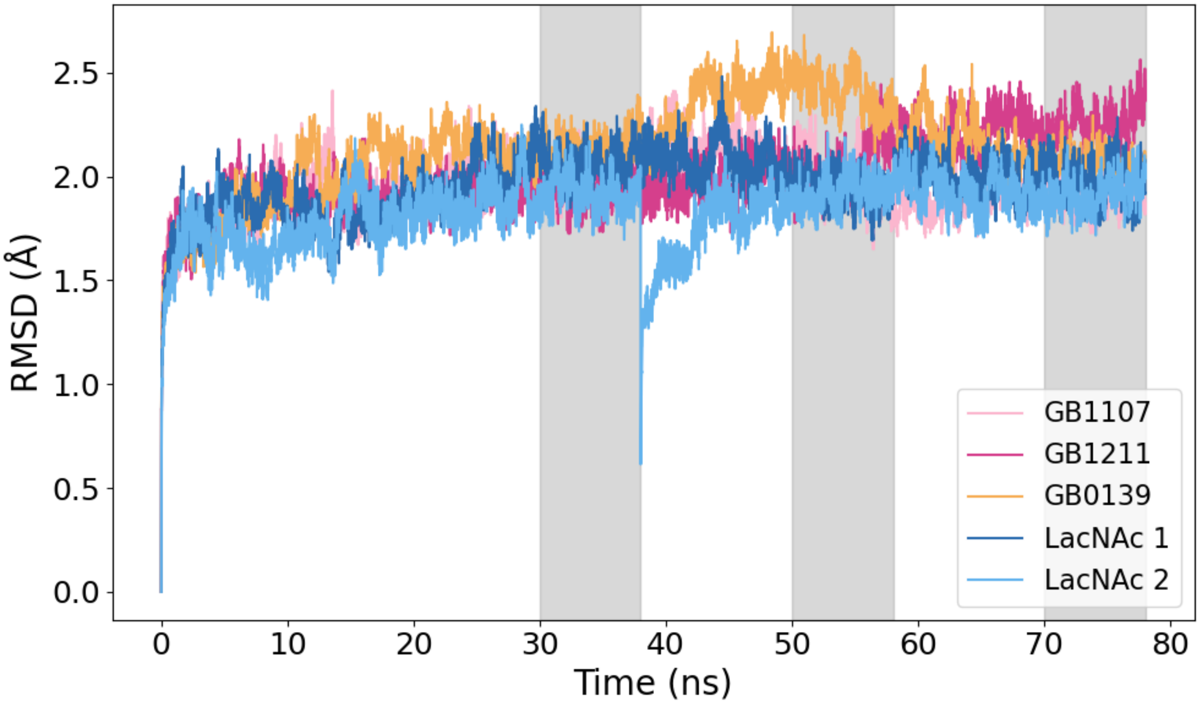

Figure S1: Root mean square deviation (RMSD) of the protein atoms as a function of simulation time.

Table S2: Flat-bottom harmonic restraint settings for GB0139, including the atom index, inner (r_i), outer (r_o) radii and force constant (k_{flat}) when not being scaled down.

GB0139

	Restraint 1	Restraint 2	Restraint 3	Restraint 4	Restraint 5
Atom Index	2295, 723	2301, 997	2270, 2044	2259, 1162	2259, 1162
r_i, r_o in Å	2.60, 3.40	2.60, 3.80	3.25, 5.50	2.50, 3.50	3.00, 6.25
k_{flat} in kcal/mol	40	40	40	40	40

Table S3: Flat-bottom harmonic restraint settings for GB1211 and GB1107, including the atom index, inner (r_i), outer (r_o) radii and force constant (k_{flat}) when not being scaled down.

GB1211

	Restraint 1	Restraint 2	Restraint 3	Restraint 4
Atom Index	2275, 1124	2257, 723	2237, 2044	2263, 997
r_i, r_o in Å	2.50, 5.25	2.60, 3.20	2.50, 5.00	2.60, 3.60
k_{flat} in kcal/mol	40	40	40	40

GB1107

	Restraint 1	Restraint 2	Restraint 3	Restraint 4
Atom Index	2275, 1124	2257, 723	2237, 2044	2263, 997
r_i, r_o in Å	2.50, 5.25	2.60, 3.40	2.50, 5.00	2.60, 3.60
k_{flat} in kcal/mol	40	40	40	40

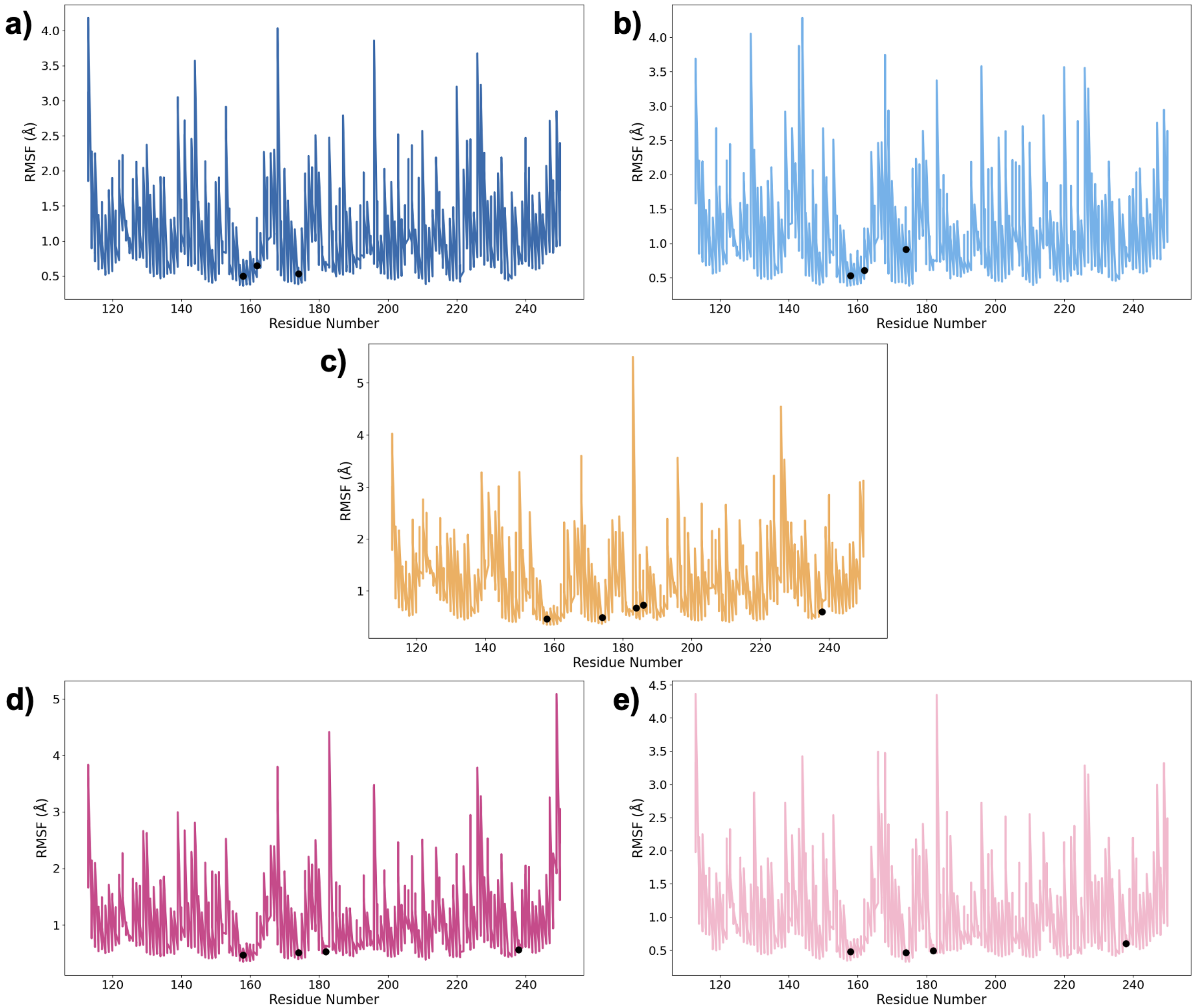

Figure S2: Root mean square fluctuation (RMSF) of a) LacNAc 1, b) LacNAc 2, c) GB0139, d) GB1211, and e) GB1107 averaged over the 78 ns simulation time. The anchor atoms used in the flat-bottom harmonic restraints are denoted with black markers.

Table S4: Harmonic restraint setting between the center of mass of each ligand and the binding pocket, with a distance (r) and a force constant (k_{harm}). The protein selection for the restraint included the following atom indices: 496-500, 719-723, 752, 755-758, 783-789, 836, 841-845, 961, 962, 967-969, 994-997, 1097-1099, 1103-1112, 1124, 1153, 1158-1162, 1190-1194

	LacNAc 1	LacNAc 2	GB1211	GB1107	GB0139
ligand index	2231-2281	2231-2281	2231-2278	2231-2279	2231-2305
r in Å	4.62	4.28	3.7	3.94	3.5
k_{harm} in kcal/mol	15	15	15	15	15

Table S5: Enumeration of all the thermodynamic states simulated for the ligands in free state (i.e., in water). The scaling factor to turn down electrostatic (λ_{ele}) and van der Waals (λ_{vdw}) are given in each case.

States	λ_{ele}	λ_{vdw}
1	1	1
2	0.9	1
3	0.8	1
4	0.7	1
5	0.6	1
6	0.5	1
7	0.4	1
8	0.3	1
9	0.2	1
10	0.1	1
11	0	1
12	0	0.975
13	0	0.95
14	0	0.9
15	0	0.85
16	0	0.8
17	0	0.75
18	0	0.7
19	0	0.65
20	0	0.6
21	0	0.55
22	0	0.5
23	0	0.4
24	0	0.3
25	0	0.2
26	0	0.1
27	0	0.05
28	0	0.025
29	0	0

Table S6: Enumeration of all the thermodynamic states simulated for the ligands in bound state (i.e., in Gal-3). The scaling factor to turn down electrostatic (λ_{ele}) and van der Waals (λ_{vdw}), as well as the force constant for the restraints, are given in each case.

States	λ_{ele}	λ_{ele}	k (kcal/mol)
1	1	1	0
2	0.9	1	10
3	0.8	1	20
4	0.7	1	30
5	0.6	1	40
6	0.5	1	40
7	0.4	1	40
8	0.3	1	40
9	0.2	1	40
10	0.1	1	40
11	0	1	40
12	0	0.975	40
13	0	0.95	40
14	0	0.9	40
15	0	0.85	40
16	0	0.8	40
17	0	0.75	40
18	0	0.725	40
19	0	0.7	40
20	0	0.65	40
21	0	0.625	40
22	0	0.6	40
23	0	0.55	40
24	0	0.5	40
25	0	0.4	40
26	0	0.3	40
27	0	0.2	40
28	0	0.1	40
29	0	0.05	40
30	0	0.025	40
31	0	0	40
32	0	0	20
33	0	0	5
34	0	0	15 (COM)

Table S7: BAR calculation of the free energy difference between each thermodynamic states for free and bound LacNAc 1 (three replicates R1-R3). The bootstrapping error is also given in each case.

States	Free	Error	Bound R1	Error	Bound R2	Error	Bound R3	Error
1—2	19.4357	0.0284	18.8752	0.0261	20.1577	0.0237	20.5449	0.0221
2—3	13.1220	0.0216	12.5574	0.0164	13.5109	0.0196	13.7161	0.0213
3—4	8.7172	0.0160	8.6548	0.0126	8.8727	0.0131	8.5971	0.0127
4—5	5.8230	0.0122	5.9909	0.0096	6.0772	0.0098	5.9899	0.0097
5—6	3.9072	0.0099	3.9657	0.0078	4.1493	0.0076	4.1528	0.0077
6—7	2.5863	0.0080	2.7322	0.0063	2.9662	0.0063	2.9033	0.0063
7—8	1.6751	0.0067	2.0501	0.0055	2.0961	0.0055	1.9865	0.0055
8—9	1.0279	0.0060	1.4425	0.0050	1.4480	0.0049	1.3781	0.0050
9—10	0.5908	0.0053	0.9754	0.0046	0.9884	0.0047	0.9407	0.0046
10—11	0.3062	0.0050	0.6918	0.0045	0.6623	0.0045	0.5778	0.0045
11—12	3.3257	0.0029	4.2333	0.0024	4.1739	0.0027	4.1653	0.0027
12—13	2.8214	0.0028	3.5727	0.0025	3.5933	0.0026	3.6427	0.0027
13—14	4.3888	0.0058	5.5581	0.0054	5.6429	0.0055	5.7709	0.0054
14—15	2.9660	0.0064	3.7714	0.0060	3.7590	0.0059	3.8899	0.0060
15—16	1.7130	0.0076	2.2600	0.0071	2.2460	0.0072	2.2882	0.0072
16—17	0.5565	0.0094	0.8284	0.0088	0.8064	0.0091	0.8667	0.0089
17—18	-0.6793	0.0124	-0.1413	0.0051	-0.1414	0.0051	-0.1243	0.0051
18—19	-2.4284	0.0202	-0.5180	0.0058	-0.5495	0.0059	-0.5167	0.0058
19—20	-5.8946	0.0454	-2.5831	0.0187	-2.7388	0.0187	-2.6481	0.0184
20—21	-8.4875	0.0222	-2.3797	0.0102	-2.4456	0.0098	-2.4515	0.0101
21—22	-4.7060	0.0058	-3.8815	0.0147	-3.7796	0.0138	-3.6674	0.0132
22—23	-2.5261	0.0029	-9.0998	0.0186	-9.0377	0.0194	-8.8361	0.0199
23—24	-0.0396	0.0004	-5.1100	0.0067	-5.1704	0.0066	-5.1011	0.0065
24—25	0.1386	0.0000	-2.9945	0.0049	-3.0136	0.0049	-2.9764	0.0048
25—26	0.0715	0.0000	-0.0319	0.0005	-0.0281	0.0005	-0.0287	0.0005
26—27	0.0263	0.0000	0.1793	0.0001	0.1802	0.0001	0.1791	0.0001
27—28	0.0128	0.0000	0.0841	0.0000	0.0845	0.0000	0.0841	0.0000
28—29	0.0128	0.0000	0.0291	0.0000	0.0292	0.0000	0.0291	0.0000
29—30			0.0141	0.0000	0.0141	0.0000	0.0141	0.0000
30—31			0.0141	0.0000	0.0141	0.0000	0.0141	0.0000
31—32			-0.1391	0.0023	-0.1370	0.0022	-0.1358	0.0022
32—33			-0.4029	0.0051	-0.4069	0.0051	-0.3932	0.0049
33—34			-2.4881	0.1093	-2.5483	0.1377	-3.5669	0.2497

Table S8: BAR calculation of the free energy difference between each thermodynamic states for free and bound LacNAc 2 (three replicates R1-R3). The bootstrapping error is also given in each case.

States	free	error	bound 1	error	bound 2	error	bound 3	error
1—2	18.2096	0.0275	17.4365	0.0311	17.9433	0.0233	18.4910	0.0199
2—3	12.1714	0.0205	10.9301	0.0179	11.8490	0.0193	12.2465	0.0200
3—4	8.0613	0.0149	7.4082	0.0119	7.4803	0.0135	7.5500	0.0127
4—5	5.3539	0.0116	5.0333	0.0092	4.8902	0.0090	5.0963	0.0098
5—6	3.5414	0.0092	3.1831	0.0074	3.3599	0.0069	3.4410	0.0074
6—7	2.3271	0.0076	2.0802	0.0061	2.3448	0.0059	2.3760	0.0060
7—8	1.5106	0.0066	1.4018	0.0053	1.6247	0.0050	1.6387	0.0051
8—9	0.9265	0.0058	0.8879	0.0048	1.1582	0.0045	1.1505	0.0047
9—10	0.5276	0.0054	0.6192	0.0046	0.7362	0.0045	0.7000	0.0046
10—11	0.2751	0.0051	0.3378	0.0042	0.3804	0.0044	0.4881	0.0045
11—12	3.3919	0.0028	4.3957	0.0025	4.3246	0.0025	4.3782	0.0026
12—13	2.8950	0.0028	3.6915	0.0024	3.7515	0.0026	3.6640	0.0027
13—14	4.5003	0.0059	5.7667	0.0053	5.9506	0.0057	5.5233	0.0057
14—15	3.0424	0.0065	3.9844	0.0060	3.9312	0.0063	3.8500	0.0061
15—16	1.7778	0.0076	2.3687	0.0070	2.2617	0.0071	2.3282	0.0071
16—17	0.5823	0.0096	0.9421	0.0086	0.8624	0.0090	0.8551	0.0088
17—18	-0.7019	0.0130	-0.1297	0.0052	-0.1258	0.0051	-0.1072	0.0051
18—19	-2.5131	0.0210	-0.5393	0.0059	-0.5518	0.0058	-0.5380	0.0058
19—20	-5.9883	0.0451	-2.3369	0.0169	-2.7779	0.0186	-2.4785	0.0172
20—21	-8.4341	0.0218	-2.2357	0.0096	-2.6269	0.0105	-2.3705	0.0100
21—22	-4.6949	0.0057	-3.4948	0.0125	-3.9475	0.0136	-3.7860	0.0136
22—23	-2.5209	0.0029	-8.4848	0.0220	-8.7780	0.0199	-8.6881	0.0199
23—24	-0.0392	0.0004	-5.1053	0.0069	-5.1205	0.0070	-5.0928	0.0068
24—25	0.1386	0.0000	-3.0198	0.0052	-3.0810	0.0054	-3.0257	0.0051
25—26	0.0715	0.0000	-0.0349	0.0006	-0.0371	0.0006	-0.0315	0.0007
26—27	0.0263	0.0000	0.1774	0.0001	0.1808	0.0001	0.1798	0.0001
27—28	0.0128	0.0000	0.0843	0.0000	0.0847	0.0000	0.0845	0.0000
28—29	0.0128	0.0000	0.0291	0.0000	0.0291	0.0000	0.0291	0.0000
29—30			0.0141	0.0000	0.0141	0.0000	0.0141	0.0000
30—31			0.0141	0.0000	0.0141	0.0000	0.0141	0.0000
31—32			-0.0714	0.0016	-0.0750	0.0017	-0.0738	0.0016
32—33			-0.2244	0.0037	-0.2316	0.0038	-0.2206	0.0036
33—34			-1.1151	0.0484	-1.2778	0.0551	-1.6125	0.0676

Table S9: BAR calculation of the free energy difference between each thermodynamic states for free and bound GB1211 (three replicates R1-R3). The bootstrapping error is also given in each case.

States	free	error	bound 1	error	bound 2	error	bound 3	error
1—2	9.7135	0.0163	9.1453	0.0108	9.0033	0.0110	9.0115	0.0112
2—3	6.4793	0.0142	6.7408	0.0095	6.5898	0.0094	6.5646	0.0095
3—4	4.0692	0.0116	4.9807	0.0078	4.8674	0.0077	4.8128	0.0079
4—5	2.4547	0.0092	3.6060	0.0065	3.7062	0.0065	3.5756	0.0065
5—6	1.4332	0.0074	2.6921	0.0056	2.7324	0.0059	2.7138	0.0057
6—7	0.7906	0.0062	2.1200	0.0052	1.9444	0.0054	2.0334	0.0051
7—8	0.4305	0.0053	1.5508	0.0051	1.4674	0.0053	1.4908	0.0050
8—9	0.2438	0.0047	1.0625	0.0053	1.0478	0.0054	1.0139	0.0053
9—10	0.1598	0.0044	0.6826	0.0049	0.6357	0.0047	0.6231	0.0050
10—11	0.1888	0.0042	0.4985	0.0043	0.4592	0.0042	0.4563	0.0044
11—12	4.3732	0.0032	5.7239	0.0026	5.7092	0.0026	5.7139	0.0027
12—13	3.7370	0.0032	4.9241	0.0027	4.9169	0.0027	4.9248	0.0027
13—14	5.8414	0.0064	7.8450	0.0059	7.7886	0.0057	7.7673	0.0056
14—15	4.0064	0.0071	5.5418	0.0063	5.4738	0.0063	5.5061	0.0064
15—16	2.4244	0.0083	3.5622	0.0074	3.5105	0.0074	3.5198	0.0074
16—17	0.9460	0.0105	1.6723	0.0096	1.7299	0.0093	1.6050	0.0096
17—18	-0.6950	0.0147	0.1508	0.0053	0.1800	0.0053	0.0708	0.0054
18—19	-2.9638	0.0242	-0.3293	0.0060	-0.3442	0.0061	-0.3601	0.0061
19—20	-7.3629	0.0655	-2.5239	0.0199	-2.5394	0.0200	-2.4860	0.0205
20—21	-10.1703	0.0253	-2.6922	0.0114	-2.5409	0.0108	-2.5641	0.0110
21—22	-5.5641	0.0061	-4.2646	0.0154	-4.1368	0.0155	-4.1843	0.0159
22—23	-2.9597	0.0032	-10.0486	0.0222	-10.2494	0.0254	-10.1168	0.0231
23—24	-0.0383	0.0004	-5.9652	0.0079	-6.1636	0.0083	-6.0547	0.0081
24—25	0.1718	0.0000	-3.6422	0.0063	-3.7885	0.0062	-3.6807	0.0063
25—26	0.0929	0.0000	-0.0425	0.0007	-0.0566	0.0007	-0.0489	0.0007
26—27	0.0353	0.0000	0.2246	0.0001	0.2234	0.0001	0.2222	0.0001
27—28	0.0173	0.0000	0.1098	0.0000	0.1095	0.0000	0.1093	0.0000
28—29	0.0172	0.0000	0.0389	0.0000	0.0389	0.0000	0.0389	0.0000
29—30			0.0189	0.0000	0.0189	0.0000	0.0189	0.0000
30—31			0.0188	0.0000	0.0188	0.0000	0.0188	0.0000
31—32			-0.1406	0.0022	-0.1425	0.0023	-0.1401	0.0022
32—33			-0.4029	0.0050	-0.4245	0.0051	-0.4092	0.0050
33—34			-3.0023	0.1570	-2.5713	0.1199	-2.8948	0.1375

Table S10: BAR calculation of the free energy difference between each thermodynamic states for free and bound GB1107 (three replicates R1-R3). The bootstrapping error is also given in each case.

States	free	error	bound 1	error	bound 2	error	bound 3	error
1—2	7.1891	0.0145	7.0155	0.0103	6.9008	0.0106	7.0788	0.0104
2—3	4.6067	0.0121	4.9228	0.0088	4.7480	0.0089	4.8744	0.0090
3—4	2.7959	0.0096	3.3502	0.0073	3.3121	0.0076	3.3325	0.0075
4—5	1.5760	0.0077	2.2102	0.0065	2.2628	0.0066	2.3243	0.0064
5—6	0.8089	0.0064	1.3523	0.0057	1.3421	0.0055	1.4227	0.0057
6—7	0.3599	0.0054	0.8296	0.0048	0.7446	0.0046	0.8510	0.0049
7—8	0.0940	0.0048	0.5719	0.0042	0.5364	0.0042	0.6145	0.0043
8—9	-0.0208	0.0043	0.4313	0.0037	0.4037	0.0037	0.4529	0.0038
9—10	0.0052	0.0040	0.4243	0.0034	0.3604	0.0035	0.4237	0.0036
10—11	0.1398	0.0039	0.5408	0.0033	0.4925	0.0035	0.4803	0.0036
11—12	4.5679	0.0032	5.8988	0.0027	5.8590	0.0027	5.8862	0.0027
12—13	3.8990	0.0032	5.1369	0.0026	5.0143	0.0028	5.0549	0.0028
13—14	6.0945	0.0067	8.1571	0.0057	7.9020	0.0060	8.0458	0.0061
14—15	4.1686	0.0073	5.6942	0.0064	5.6139	0.0065	5.6517	0.0067
15—16	2.5222	0.0084	3.6411	0.0076	3.6509	0.0077	3.6362	0.0076
16—17	0.9999	0.0105	1.7581	0.0095	1.7503	0.0096	1.6859	0.0099
17—18	-0.6758	0.0150	0.1774	0.0055	0.1718	0.0055	0.1128	0.0054
18—19	-3.0387	0.0256	-0.3321	0.0063	-0.2606	0.0061	-0.3330	0.0062
19—20	-7.9494	0.0743	-2.6159	0.0206	-2.5849	0.0222	-2.6236	0.0214
20—21	-10.4220	0.0253	-2.6180	0.0108	-2.7698	0.0113	-2.7535	0.0113
21—22	-5.7456	0.0061	-4.2826	0.0155	-4.4308	0.0156	-4.1507	0.0148
22—23	-3.0698	0.0032	-10.3567	0.0237	-10.2814	0.0228	-10.0319	0.0247
23—24	-0.0400	0.0004	-6.1464	0.0084	-6.1091	0.0083	-6.0523	0.0079
24—25	0.1775	0.0001	-3.7814	0.0068	-3.7571	0.0066	-3.7932	0.0067
25—26	0.0958	0.0000	-0.0529	0.0007	-0.0524	0.0007	-0.0569	0.0008
26—27	0.0363	0.0000	0.2306	0.0001	0.2303	0.0001	0.2313	0.0001
27—28	0.0178	0.0000	0.1129	0.0000	0.1129	0.0000	0.1129	0.0000
28—29	0.0177	0.0000	0.0400	0.0000	0.0400	0.0000	0.0400	0.0000
29—30			0.0194	0.0000	0.0194	0.0000	0.0194	0.0000
30—31			0.0194	0.0000	0.0194	0.0000	0.0194	0.0000
31—32			-0.1299	0.0022	-0.1294	0.0021	-0.1274	0.0021
32—33			-0.3810	0.0049	-0.3756	0.0049	-0.3828	0.0049
33—34			-3.2065	0.1832	-4.9936	0.5497	-2.9482	0.1615

Table S11: BAR calculation of the free energy difference between each thermodynamic states for free and bound GB0139 (three replicates R1-R3). The bootstrapping error is also given in each case.

Steps	free	error	bound 1	error	bound 2	error	bound 3	error
1—2	14.7340	0.0252	15.4160	0.0243	15.8802	0.0247	16.5885	0.0199
2—3	9.5537	0.0190	10.1236	0.0157	9.7972	0.0162	10.9286	0.0182
3—4	5.8986	0.0146	6.8494	0.0130	6.1361	0.0119	6.9562	0.0114
4—5	3.5577	0.0111	4.3178	0.0094	3.9795	0.0087	4.6039	0.0098
5—6	2.1211	0.0091	2.7190	0.0074	2.6465	0.0071	2.7633	0.0080
6—7	1.1907	0.0077	1.7709	0.0063	1.7753	0.0063	1.8742	0.0069
7—8	0.6297	0.0068	1.1166	0.0058	1.2347	0.0056	1.2219	0.0058
8—9	0.3134	0.0061	0.6721	0.0051	0.8117	0.0053	0.7335	0.0052
9—10	0.1940	0.0056	0.4821	0.0046	0.6016	0.0050	0.5167	0.0051
10—11	0.2291	0.0053	0.5204	0.0045	0.5723	0.0048	0.5050	0.0047
11—12	5.9947	0.0039	7.6507	0.0037	7.7086	0.0034	7.7134	0.0035
12—13	5.1090	0.0039	6.4205	0.0036	6.5172	0.0033	6.5605	0.0037
13—14	7.9699	0.0078	10.0030	0.0072	9.9152	0.0071	10.0603	0.0073
14—15	5.1804	0.0081	6.9681	0.0078	6.9046	0.0079	6.8092	0.0076
15—16	3.1234	0.0096	4.3112	0.0091	4.3842	0.0092	4.2642	0.0089
16—17	1.3737	0.0122	1.8242	0.0122	1.8219	0.0124	1.7716	0.0121
17—18	-0.5926	0.0164	-0.0145	0.0066	-0.0609	0.0067	-0.1063	0.0067
18—19	-3.1982	0.0282	-0.6911	0.0077	-0.7080	0.0076	-0.7266	0.0076
19—20	-9.4386	0.1288	-3.9859	0.0281	-3.8196	0.0269	-4.0398	0.0286
20—21	-14.1473	0.0372	-3.9679	0.0143	-3.9186	0.0146	-4.1330	0.0146
21—22	-7.9262	0.0076	-6.2409	0.0202	-6.1840	0.0202	-6.3938	0.0201
22—23	-4.2394	0.0038	-14.5799	0.0319	-14.8787	0.0374	-14.8117	0.0361
23—24	-0.0580	0.0005	-8.3879	0.0084	-8.6945	0.0089	-8.4210	0.0083
24—25	0.2425	0.0001	-4.8193	0.0059	-4.9231	0.0062	-4.8259	0.0060
25—26	0.1293	0.0000	-0.0293	0.0007	-0.0353	0.0007	-0.0518	0.0007
26—27	0.0487	0.0000	0.3015	0.0001	0.3000	0.0001	0.3036	0.0001
27—28	0.0238	0.0000	0.1487	0.0000	0.1478	0.0000	0.1488	0.0000
28—29	0.0237	0.0000	0.0535	0.0000	0.0535	0.0000	0.0535	0.0000
29—30			0.0260	0.0000	0.0260	0.0000	0.0260	0.0000
30—31			0.0260	0.0000	0.0260	0.0000	0.0260	0.0000
31—32			-0.1809	0.0025	-0.1795	0.0025	-0.1770	0.0025
32—33			-0.5128	0.0056	-0.5148	0.0057	-0.5093	0.0056
33—34			-4.1476	0.2937	-4.3660	0.3486	-4.2553	0.2993

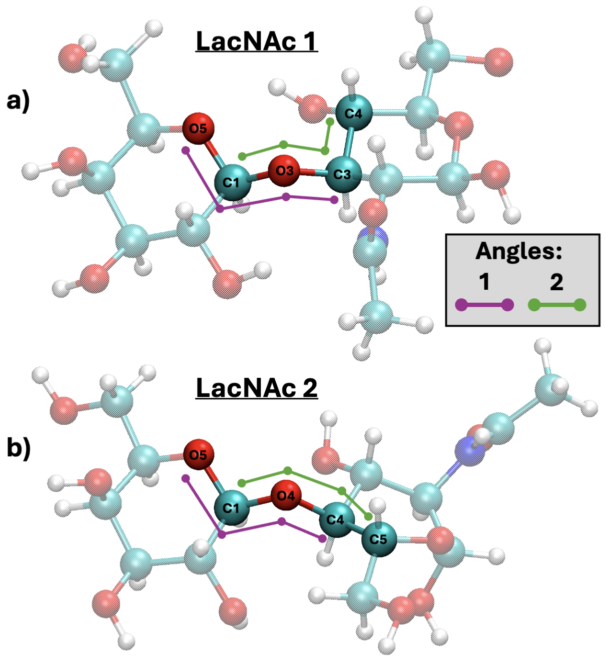

Figure S3: Representation of the two glycosidic angles (highlighted with magenta and green lines) used to describe binding pose of a) LacNAc Type 1 and b) LacNAc type 2.

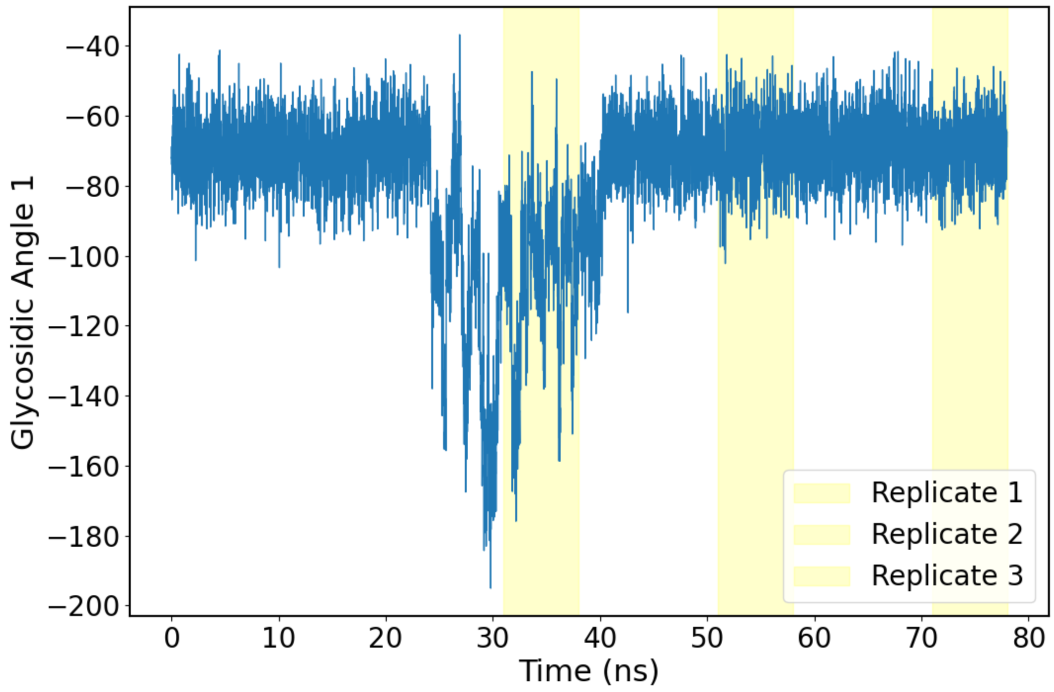

Figure S4: Dihedral angle 1 for LacNAc 1 over time, showing the variations between replicates.

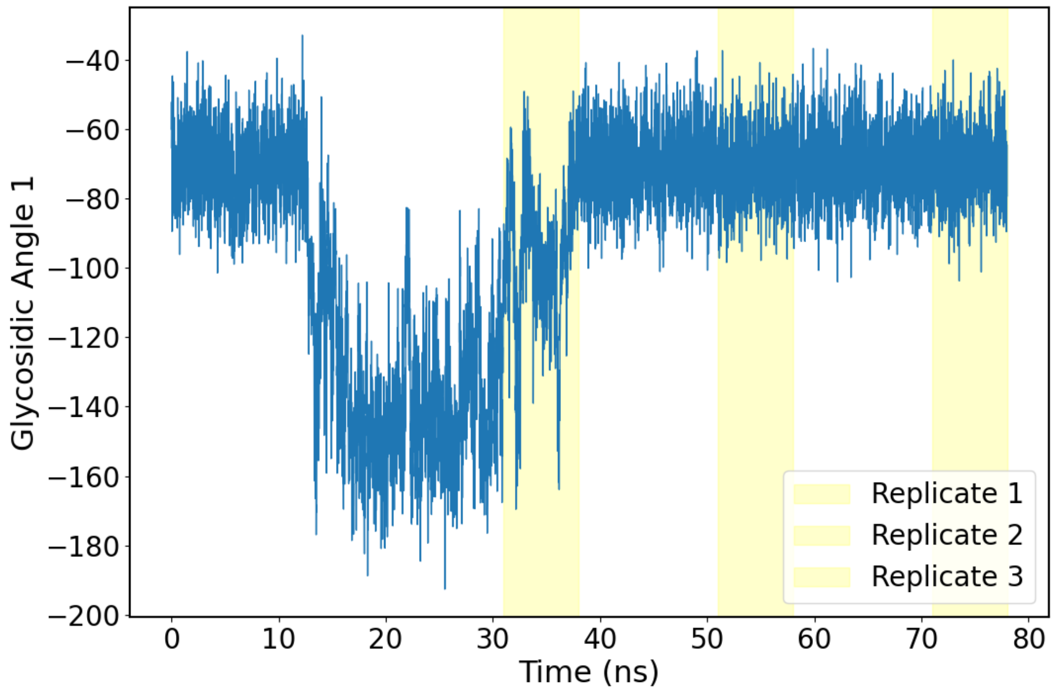

Figure S5: Dihedral angle 2 for LacNAc 1 over time, showing the variations between replicates.

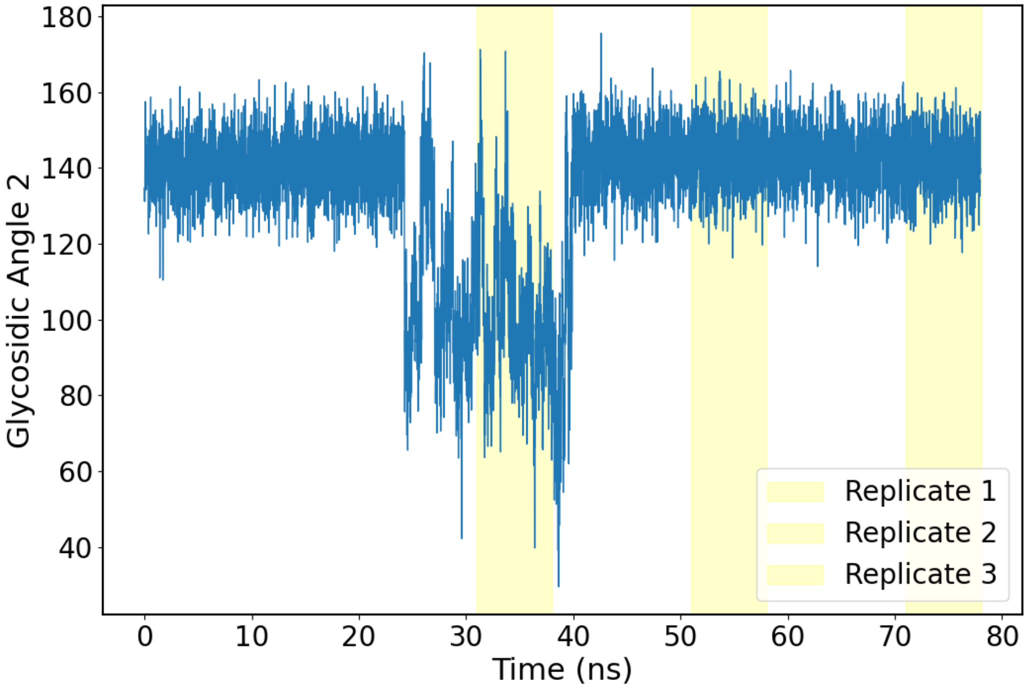

Figure S6: Dihedral angle 1 for LacNAc 2 over time, showing the variations between replicates.

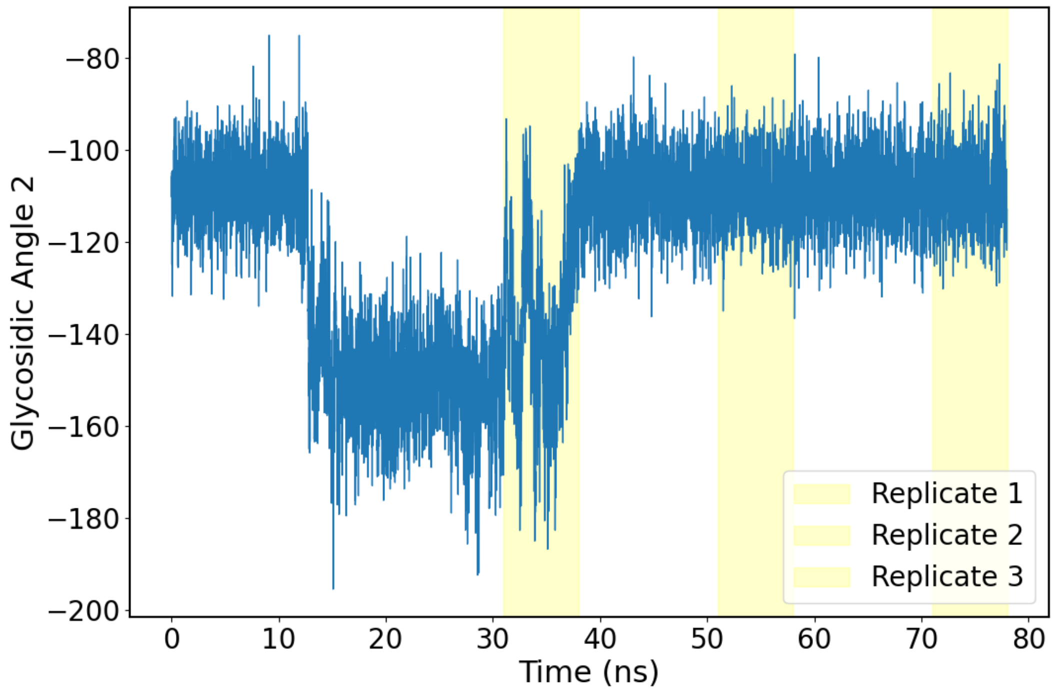

Figure S7: Dihedral angle 2 for LacNAc 2 over time, showing the variations between replicates.

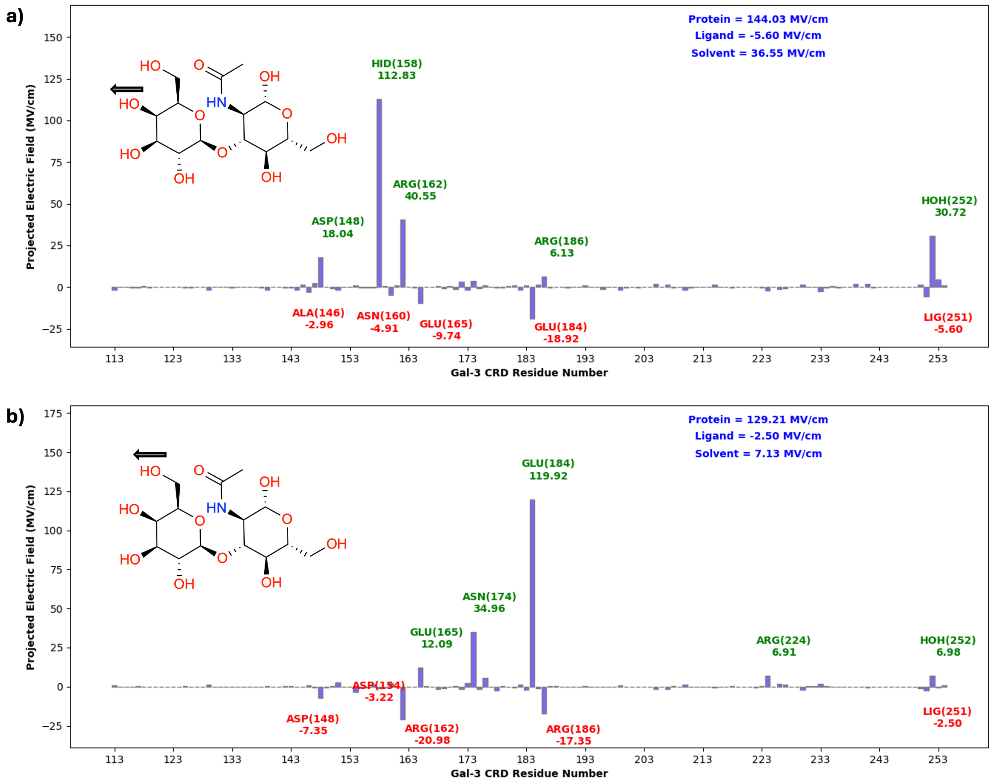

Figure S8: Electric field contributions for LacNAc type 1 projected along the a) O4-H bond and b) O6-H bond. Residues 113-250 comprise the protein amino acids, residue 251 is the ligand contribution, and the solvent is comprised of water, sodium, and chlorine which are residues 252, 253, and 354 respectively.

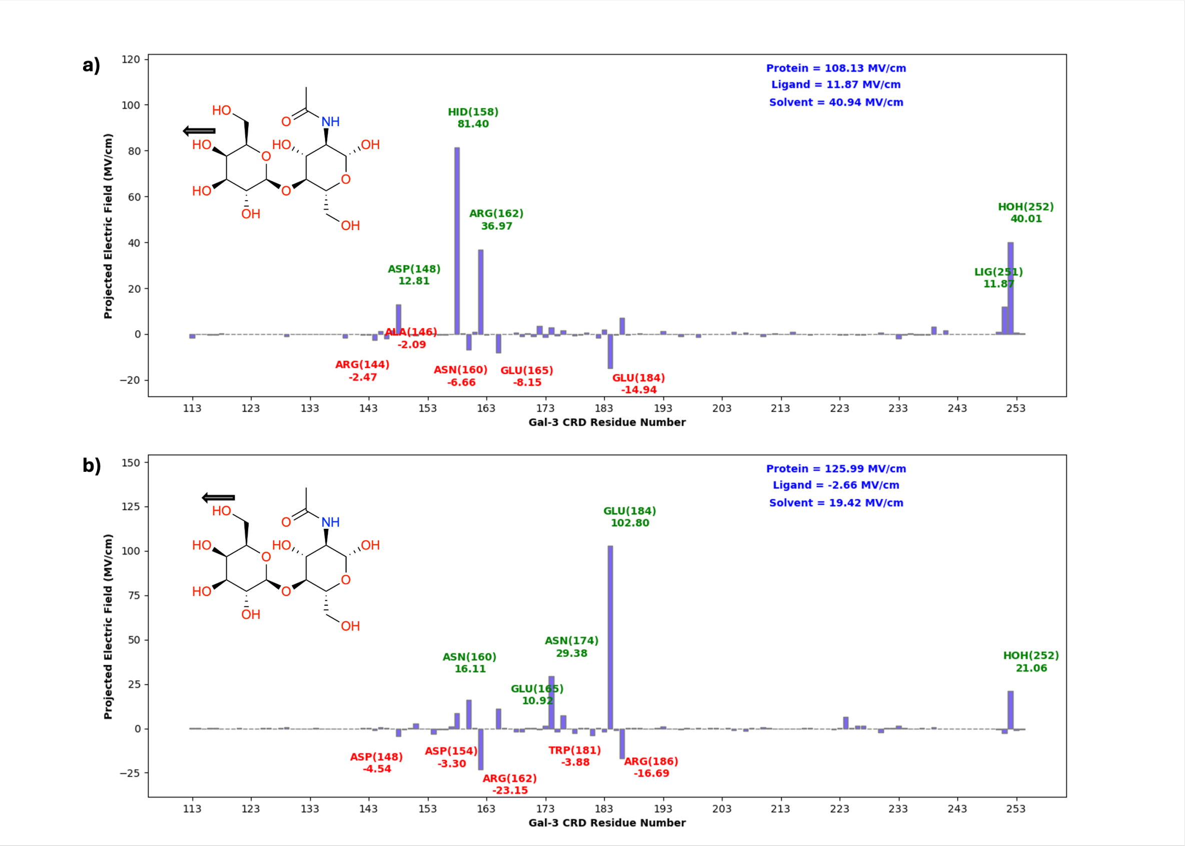

Figure S9: Electric field contributions for LacNAc type 2 projected along the a) O4-H bond and b) O6-H bond. Residues 113-250 comprise the protein amino acids, residue 251 is the ligand contribution, and the solvent is comprised of water, sodium, and chlorine which are residues 252, 253, and 354 respectively.

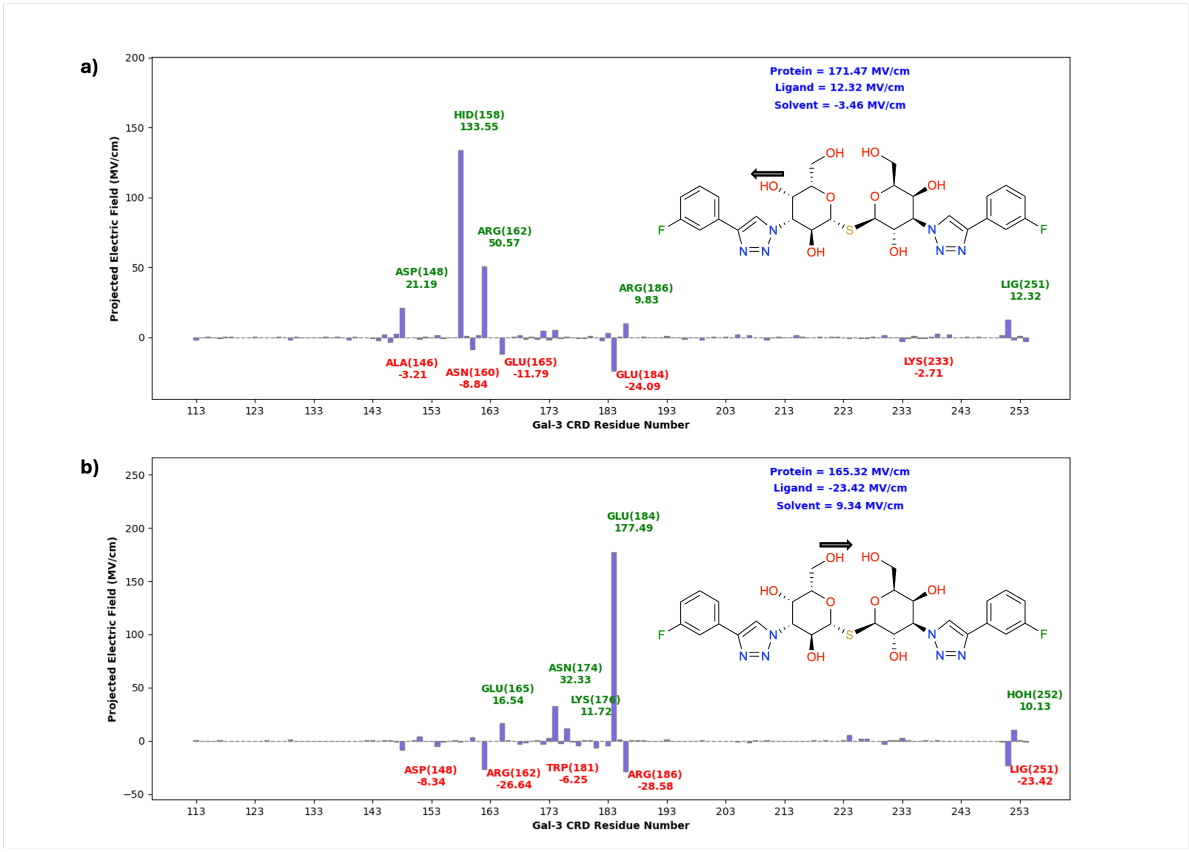

Figure S10: Electric field contributions for GB0139 projected along the a) O4-H bond and b) O6-H bond. Residues 113-250 comprise the protein amino acids, residue 251 is the ligand contribution, and the solvent is comprised of water, sodium, and chlorine which are residues 252, 253, and 354 respectively.

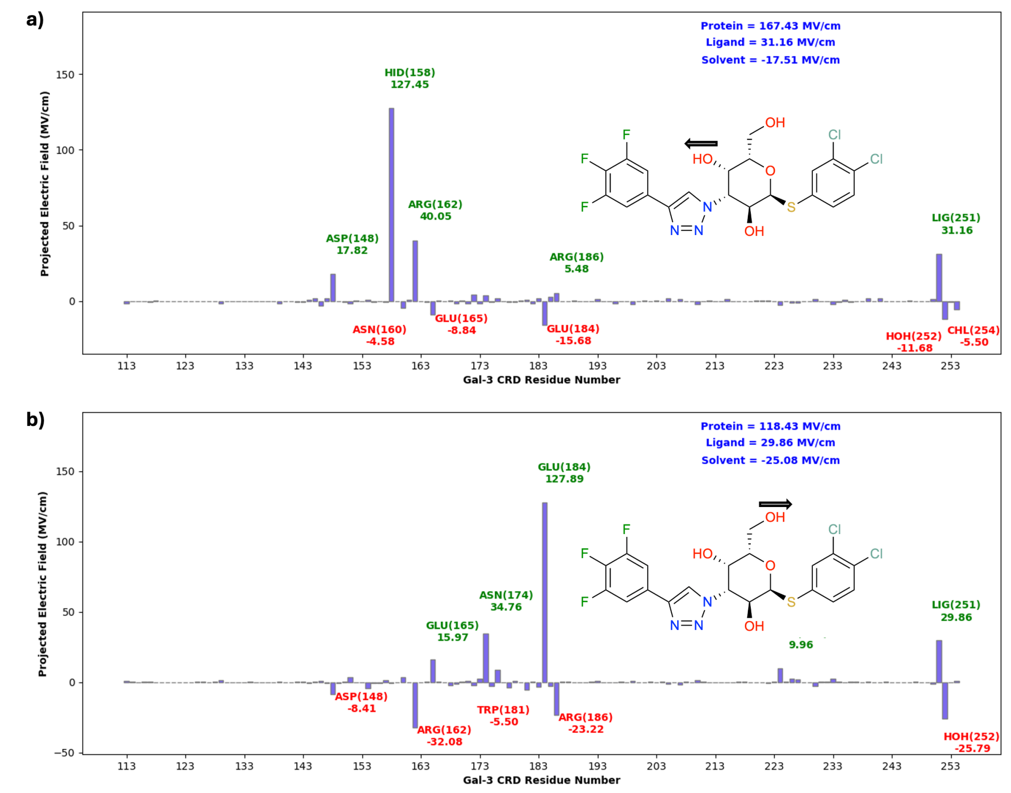

Figure S11: Electric field contributions for GB1107 projected along the a) O4-H bond and b) O6-H bond. Residues 113-250 comprise the protein amino acids, residue 251 is the ligand contribution, and the solvent is comprised of water, sodium, and chlorine which are residues 252, 253, and 354 respectively.

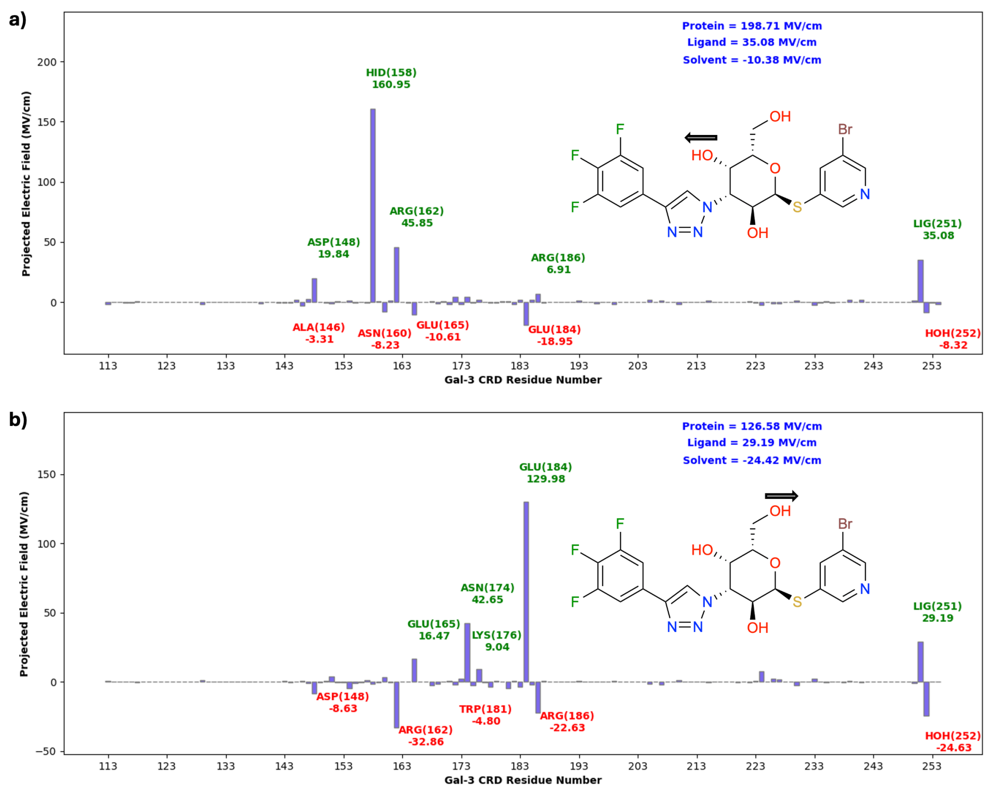

Figure S12: Electric field contributions for GB1107 projected along the a) O4-H bond and b) O6-H bond. Residues 113-250 comprise the protein amino acids, residue 251 is the ligand contribution, and the solvent is comprised of water, sodium, and chlorine which are residues 252, 253, and 354 respectively.

Table S12: Free energy differences between thermodynamic states that 'disappear' the ligand from water. As described in Methods, the electrostatics (van der Waals) interactions were annihilated over 10 (18) states. The free energy differences between consecutive states were calculated with BAR, summed, and presented as $\Delta G_{\text{F-ele}}$ and $\Delta G_{\text{F-vdw}}$. The total $\Delta G_{\text{F-total}} = \Delta G_{\text{F-ele}} + \Delta G_{\text{F-vdw}}$.

Energy Diff. (kcal/mol)	LacNAc 1	LacNAc 2	GB1211	GB1107	GB0139
$\Delta G_{\text{F-ele}}$	57.19	52.90	25.96	17.55	38.42
$\Delta G_{\text{F-vdw}}$	-8.73	-8.44	-8.09	-8.34	-10.38
$\Delta G_{\text{F-total}}$	48.46	44.46	17.87	9.21	28.04

Table S13: Free energy differences between thermodynamic states that 'disappear' the ligand from the protein. As described in Methods, the electrostatics (van der Waals) interactions were annihilated over 10 (20) states. The free energy differences between consecutive states were calculated with BAR, summed, and presented as $\Delta G_{\text{B-ele}}$ and $\Delta G_{\text{B-vdw}}$. The total $\Delta G_{\text{B-total}} = \Delta G_{\text{B-ele}} + \Delta G_{\text{B-vdw}} + \Delta G_{\text{rest-2}}$.

Energy Diff. (kcal/mol)		LacNAc1			LacNAc2	
Replicates	R1	R2	R3	R1	R2	R3
$\Delta G_{\text{B-ele+rest-1}}$	57.94	60.93	60.79	49.32	51.77	53.18
$\Delta G_{\text{B-vdw}}$	-6.20	-6.36	-5.41	-3.91	-5.64	-5.20
$\Delta G_{\text{rest-2}}$	-3.03	-3.09	-4.10	-1.41	-1.58	-1.91
$\Delta G_{\text{B-total}}$	48.71	51.48	51.29	43.99	44.54	46.07
Energy Diff. (kcal/mol)		GB1211			GB1107	
Replicates	R1	R2	R3	R1	R2	R3
$\Delta G_{\text{B-ele+rest-1}}$	33.08	32.45	32.30	21.65	21.10	21.86
$\Delta G_{\text{B-vdw}}$	0.32	-0.10	0.02	0.70	0.14	0.70
$\Delta G_{\text{rest-2}}$	-3.55	-3.14	-3.44	-3.72	-5.50	-3.46
$\Delta G_{\text{B-total}}$	29.86	29.21	28.87	18.63	15.74	19.10
Energy Diff. (kcal/mol)		GB0139				
Replicates	R1	R2	R3			
$\Delta G_{\text{B-ele+rest-1}}$	43.99	43.44	46.69			
$\Delta G_{\text{B-vdw}}$	-4.98	-5.42	-5.77			
$\Delta G_{\text{rest-2}}$	-4.84	-5.06	-4.94			
$\Delta G_{\text{B-total}}$	34.16	32.96	35.98			

Table S14: Electric fields projected onto specific bonds in LacNAc 1, LacNAc 2 and GB0139, in MV/cm. Values reported are averages over 7 ns MD trajectories of each molecule bound to Gal-3, for each replicate. Positive projections mean that the electric field is oriented from the first atom (O or C) to the second (H, X or F).

	LacNAc1			LacNAc2			GB0139		
Replicates	R1	R2	R3	R1	R2	R3	R1	R2	R3
O_2−H prot.	6.3	8.4	8.5	1.7	7.2	3.5	-0.7	-2.0	-5.2
O_2−H sol.	105.7	143.6	148.0	137.1	146.1	135.6	155.9	157.7	159.4
O_4−H prot.	112.4	158.5	161.2	11.1	152.9	160.4	170.7	171.8	171.9
O_4−H sol.	55.5	26.9	27.3	114.3	4.5	1.6	-2.4	-4.4	-3.7
O_6−H prot.	89.2	148.4	150.0	79.1	150.2	148.6	164.6	164.9	166.4
O_6−H sol.	21.1	1.6	-1.3	75.6	-6.1	-5.9	6.62	11.0	10.4
C−F prot.	-	-	-	-	-	-	-4.4	-10.7	-11.0
C−F sol.	-	-	-	-	-	-	-7.1	-1.2	-3.3

Table S15: Electric fields projected onto specific bonds in GB1211 and GB1107, in MV/cm. Values reported are averages over 7 ns MD trajectories of each molecule bound to Gal-3, for each replicate. Positive projections mean that the electric field is oriented from the first atom (O or C) to the second (H, X or F).

	GB1211			GB1107		
Replicates	R1	R2	R3	R1	R2	R3
O_2−H prot.	0.0	-0.8	-1.5	-3.9	-4.0	-11.2
O_2−H sol.	155.6	154.3	160.7	187.7	183.3	201.1
O_4−H prot.	199.4	198.7	198.0	165.2	167.9	169.1
O_4−H sol.	-9.0	-8.2	-13.9	-14.8	-17.5	-20.2
O_6−H prot.	135.9	124.7	119.1	126.8	115.0	113.5
O_6−H sol.	-28.7	-25.4	-19.1	-27.6	-23.5	-24.2
C−X prot.	2.5	8.1	-0.3	-12.2	-11.0	-7.4
C−X sol.	-5.7	-9.6	-2.4	-8.0	-6.0	-10.4
C−F prot.	-20.6	-22.5	-22.5	-21.5	-19.6	-8.0
C−F sol.	1.9	3.4	2.3	0.3	-1.3	-10.5
